# Diabetes-Modifying Antirheumatic Drugs: The Roles of DMARDs as Glucose-Lowering Agents

**DOI:** 10.3390/medicina58050571

**Published:** 2022-04-21

**Authors:** Marco Infante, Nathalia Padilla, Rodolfo Alejandro, Massimiliano Caprio, David Della-Morte, Andrea Fabbri, Camillo Ricordi

**Affiliations:** 1 Clinical Cell Transplant Program (CCTP), Diabetes Research Institute, University of Miami Miller School of Medicine, 1450 NW 10th Ave, Miami, FL 33136, USA; ralejand@med.miami.edu (R.A.); cricordi@med.miami.edu (C.R.); 2 Department of Systems Medicine, Diabetes Research Institute Federation (DRIF), University of Rome Tor Vergata, Via Montpellier 1, 00133 Rome, Italy; andrea.fabbri@uniroma2.it; 3 Section of Endocrinology, UniCamillus, Saint Camillus International University of Health Sciences, Via di Sant’Alessandro 8, 00131 Rome, Italy; 4 Network of Immunity in Infection, Malignancy and Autoimmunity (NIIMA), Universal Scientific Education and Research Network (USERN), Via Cola di Rienzo 28, 00192 Rome, Italy; 5 Network of Immunity in Infection, Malignancy and Autoimmunity (NIIMA), Universal Scientific Education and Research Network (USERN), Colonia Centroamérica L-823, Managua 14048, Nicaragua; nathalia.padilla22@gmail.com; 6 Laboratory of Cardiovascular Endocrinology, IRCCS San Raffaele Roma, Via di Val Cannuta 247, 00166 Rome, Italy; massimiliano.caprio@sanraffaele.it; 7 Department of Human Sciences and Promotion of the Quality of Life, San Raffaele Roma Open University, Via di Val Cannuta 247, 00166 Rome, Italy; david.dellamorte@uniroma2.it; 8 Department of Systems Medicine, University of Rome Tor Vergata, Via Montpellier 1, 00133 Rome, Italy; 9 Department of Neurology, Evelyn F. McKnight Brain Institute, University of Miami Miller School of Medicine, 1120 NW 14th St., Miami, FL 33136, USA

**Keywords:** beta cell, cardiovascular disease, chronic inflammatory rheumatic diseases, diabetes, disease-modifying antirheumatic drugs, DMARDs, drug repurposing, inflammation, insulin resistance, type 2 diabetes

## Abstract

Systemic inflammation represents a shared pathophysiological mechanism which underlies the frequent clinical associations among chronic inflammatory rheumatic diseases (CIRDs), insulin resistance, type 2 diabetes (T2D), and chronic diabetes complications, including cardiovascular disease. Therefore, targeted anti-inflammatory therapies are attractive and highly desirable interventions to concomitantly reduce rheumatic disease activity and to improve glucose control in patients with CIRDs and comorbid T2D. Therapeutic approaches targeting inflammation may also play a role in the prevention of prediabetes and diabetes in patients with CIRDs, particularly in those with traditional risk factors and/or on high-dose corticosteroid therapy. Recently, several studies have shown that different disease-modifying antirheumatic drugs (DMARDs) used for the treatment of CIRDs exert antihyperglycemic properties by virtue of their anti-inflammatory, insulin-sensitizing, and/or insulinotropic effects. In this view, DMARDs are promising drug candidates that may potentially reduce rheumatic disease activity, ameliorate glucose control, and at the same time, prevent the development of diabetes-associated cardiovascular complications and metabolic dysfunctions. In light of their substantial antidiabetic actions, some DMARDs (such as hydroxychloroquine and anakinra) could be alternatively termed “diabetes-modifying antirheumatic drugs”, since they may be repurposed for co-treatment of rheumatic diseases and comorbid T2D. However, there is a need for future randomized controlled trials to confirm the beneficial metabolic and cardiovascular effects as well as the safety profile of distinct DMARDs in the long term. This narrative review aims to discuss the current knowledge about the mechanisms behind the antihyperglycemic properties exerted by a variety of DMARDs (including synthetic and biologic DMARDs) and the potential use of these agents as antidiabetic medications in clinical settings.

## 1. Introduction

Type 2 diabetes (T2D) is one of the most common chronic noncommunicable diseases and represents a growing public health challenge worldwide. Poor glucose control in diabetic patients can result in the development of micro- and macrovascular complications [1], with cardiovascular disease (CVD) being the leading cause of death in such patients [2]. According to recent estimates from the International Diabetes Federation, the global diabetes prevalence is rapidly rising, with 463 million people being affected worldwide [3]. This trend is predicted to grow further in the upcoming years, with 578 million and 700 million people expected to develop diabetes by 2030 and 2045, respectively [3]. T2D accounts for approximately 90–95% of all diabetes cases and results from the gradual loss of adequate beta cell insulin secretion, frequently on the background of peripheral insulin resistance [4]. Insulin resistance by itself represents an established cardiovascular risk factor in a variety of population groups, including subjects with and without diabetes or other cardiovascular risk factors [5].

A growing body of evidence suggests that inflammation plays a pivotal role in the pathophysiology of T2D and diabetes-related cardiovascular complications [6,7]. Patients with T2D often exhibit a state of chronic low-grade systemic inflammation characterized by a marked increase in circulating levels of acute-phase proteins and proinflammatory chemokines, cytokines, and adipokines—including C-reactive protein (CRP), tumor necrosis factor (TNF)-α, interleukin (IL)-1, IL-6, visfatin, and resistin—accompanied by a decrease in anti-inflammatory and insulin-sensitizing adipokines such as adiponectin and omentin [8,9,10,11,12,13,14]. This proinflammatory state even occurs prior to the onset of hyperglycemia (during the prediabetic stage) and is involved in the subsequent development of T2D [8]. Therefore, more recently, T2D has increasingly been regarded as an inflammatory disease, with chronic low-grade systemic inflammation and islet inflammation being linked to insulin resistance and beta cell dysfunction, respectively [15,16,17,18]. On the one hand, three major sources of proinflammatory cytokines have been suggested to affect beta cell function, insulin secretion, and/or insulin sensitivity in T2D: (i) from islet cells; (ii) from increased numbers of islet infiltrating macrophages/immune cells; (iii) from inflamed white adipose tissue, as a consequence of obesity, physical inactivity, and adipose tissue dysfunction [8,14,19]. On the other hand, patients with chronic inflammatory rheumatic diseases (CIRDs) often exhibit greater insulin resistance and a higher frequency of features of the metabolic syndrome as compared with controls [20,21,22,23], as well as an increased risk of comorbid or incident diabetes [22,24,25,26,27,28,29,30] and higher cardiovascular morbidity and mortality [31,32,33,34,35,36,37,38]. Although CVD represents a leading cause of morbidity and mortality in CIRD patients [31,38,39,40], risk factors for CVD in this population are not yet entirely clear. The coexistence of systemic inflammation and traditional risk factors (e.g., hypertension, obesity, impaired fasting glucose, T2D, hypercholesterolemia, physical inactivity, and smoking) partly accounts for the excess cardiovascular risk and cardiovascular mortality observed in patients with CIRDs [22,35,41,42,43,44,45,46,47,48]. Thus, systemic inflammation (alone or in combination with traditional risk factors and/or corticosteroid use) likely represents a major contributor to insulin resistance, metabolic syndrome, development of T2D, and increased cardiovascular morbidity and mortality in patients with CIRDs [23,27,34,43,47,49].

In light of these preliminary remarks, targeted anti-inflammatory therapies represent attractive and highly desirable intervention strategies to prevent or treat T2D and its complications, and to concurrently control rheumatic disease activity in patients with CIRDs [50]. This narrative review aims to discuss the current knowledge about the antihyperglycemic properties exerted by a variety of disease-modifying antirheumatic drugs (DMARDs) and the potential use of these agents as antidiabetic medications in clinical settings.

## 2. Disease-Modifying Antirheumatic Drugs (DMARDs)

DMARDs are a class of immunomodulatory and immunosuppressive agents used for the treatment of CIRDs, such as rheumatoid arthritis (RA), systemic lupus erythematosus (SLE), and Sjögren’s syndrome (SS) [51]. DMARDs can be classified into two main categories, namely synthetic DMARDs and biologic DMARDs.

Synthetic DMARDS are classified as either (i) conventional synthetic DMARDs (csDMARDs), including methotrexate, hydroxychloroquine, sulfasalazine, and leflunomide or (ii) targeted synthetic DMARDs (tsDMARDs) [51,52]. Targeted synthetic DMARDs are small molecules targeting a particular molecular structure and include phosphodiesterase inhibitors and kinase inhibitors, such as apremilast and Janus kinase (JAK) inhibitors (i.e., JAKIs: tofacitinib, baricitinib, upadacitinib, and filgotinib) [52].

Biologic DMARDs (bDMARDs) are highly specific agents targeting a specific pathway of the immune system. These drugs were introduced in the early 1990s and are generally prescribed in the case of conventional DMARD therapy failure due to ongoing disease activity, or clinical and/or radiographic disease progression. Biologic DMARDs include TNF inhibitors (e.g., etanercept, adalimumab, infliximab, golimumab, and certolizumab pegol) and biologics that target other cytokines and molecules or receptors, such as anakinra, canakinumab, tocilizumab, sarilumab, and abatacept [51].

## 3. Conventional Synthetic DMARDs (csDMARDs)

### 3.1. Methotrexate (MTX)

Methotrexate (MTX) is generally the first choice for the treatment of RA, psoriatic arthritis (PsA), and other forms of inflammatory arthritis. Moreover, MTX enhances the effects of several biologic agents in RA [53]. MTX mainly acts as an antimetabolite and antifolate, since it inhibits the enzyme dihydrofolate reductase (DHFR), which catalyzes a reduction in folate and 7,8-dihydrofolate to 5,6,7,8-tetrahydrofolate, and is involved in purine and thymidylate synthesis. As a result, MTX inhibits DNA synthesis and cell proliferation [54].

Multiple mechanisms underlie the anti-inflammatory and immunosuppressive actions of MTX, such as the inhibition of purine and pyrimidine synthesis, nitric oxide production, transmethylation reactions, and translocation of the transcription factor nuclear factor kappa B (NF-κB) to the nucleus [53]. However, little is known about the exact role of MTX in insulin resistance and existing studies have yielded conflicting findings.

A small intervention study conducted in nondiabetic patients with RA and PsA who received 6-month MTX treatment reported a significant 0.3% reduction in glycated hemoglobin (HbA1c), although homeostasis model assessment of insulin resistance (HOMA-IR) remained unaltered [55]. A recent meta-analysis found that RA patients on MTX treatment had a lower risk of developing T2D as compared with RA patients who were not exposed to MTX [56]. Mantravadi et al. [57] recently conducted a large retrospective cohort study in patients with PsA, RA, or ankylosing spondylitis (AS) who had comorbid diabetes and newly initiated either a TNF inhibitor, MTX, or metformin (patients who received metformin served as positive controls). The authors found that initiation of a TNF inhibitor or MTX was associated with a decrease in HbA1c that was around half as much (~0.4 units) as the decrease observed after metformin initiation (~0.8 units). In a cohort with a HbA1c value of  ≥7%, TNF inhibitor initiators showed less of a decline in HbA1c as compared with MTX initiators (after adjustment for sex, age, baseline HbA1c, and comorbidities) [57]. Baker et al. [29] recently conducted a cohort study aimed to determine associations between inflammatory disease activity and incident diabetes mellitus among 1866 patients with RA from the Veteran’s Affairs Rheumatoid Arthritis Registry (without prevalent diabetes mellitus at enrollment). Multivariable Cox proportional hazard models revealed that high disease activity score (DAS28)-CRP, obesity, older age, and male sex were associated with a significantly higher risk for incident diabetes mellitus, whereas smoking habit and MTX use were protective (associated with a significantly lower risk of diabetes mellitus). Conversely, the authors did not identify significant associations with other RA therapies such as hydroxychloroquine, TNF inhibitors, or abatacept independent of other factors such as disease activity [29]. These findings are surprising, as several cytokines and chemokines evaluated in the study were independently associated with the incidence of diabetes mellitus, including IL-1, IL-6, macrophage-derived chemokine (MDC), monocyte chemoattractant protein-1 (MCP-1), MCP-4, macrophage inflammatory protein (MIP)-1β, and MIP-3α. These associations were independent of the clinical disease activity measured by DAS28-CRP. The authors hypothesized that the significant association of MTX use with lower risk of diabetes could be attributed to causal reduction in risk that may be independent of the MTX effects on measures of disease activity. Nevertheless, the authors also specified that there may be residual confounding at play since patients with hepatic steatosis could have been both less likely to use MTX [29] (due to potential MTX hepatotoxicity) [58] and at higher risk of developing diabetes [29].

The exact mechanisms through which MTX may influence glucose homeostasis remain unclear. MTX may offer long-lasting beneficial metabolic effects beyond its anti-inflammatory effects [56,59]. It has been hypothesized that MTX improves glucose homeostasis, as intracellular MTX polyglutamate inhibits 5-aminoimidazole-4-carboxamide ribonucleotide formyltransferase. This leads to the accumulation of the substrate aminoimidazole carboxamide ribonucleotide (AICAR), with its metabolites inhibiting adenosine deaminase and adenosine monophosphate (AMP) deaminase [56,60]. In turn, intracellular concentrations of AICAR monophosphate (ZMP) and AMP increase [56,61]. ZMP then directly activates the AMP-activated protein kinase (AMPK) [62]. AMPK activation is known to ameliorate glucose homeostasis by decreasing hepatic glucose production and increasing peripheral insulin sensitivity [63,64]. In this regard, MTX may exert metformin-like properties [65,66]. Indeed, Pirkmajer et al. [67] showed that MTX enhanced AICAR-stimulated AMPK activation and potentiated glucose uptake and lipid oxidation in cultured myotubes and in skeletal muscle isolated from mice, as a likely consequence of reduced ZMP clearance. However, if the aforementioned mechanism is also present in humans is still uncertain. Indeed, a small, 6-month intervention study conducted by Perdan-Pirkmajer et al. [55] in nondiabetic patients with RA and PsA showed that reductions in HbA1c values observed after MTX therapy were not associated with increased erythrocyte ZMP or urine AICAR concentrations, and concluded that MTX probably did not produce a chronic increase in erythrocyte ZMP concentration and urinary AICAR excretion in such patients.

Therefore, future mechanistic studies are warranted to better understand the mechanisms of actions underlying the antihyperglycemic properties exerted by MTX. Large randomized controlled trials are also needed to establish the existence of a true causal relationship between MTX use and reduced risk of diabetes mellitus in patients with CIRDs.

### 3.2. Hydroxychloroquine (HCQ)

Antimalarials such as chloroquine (CQ) and its hydroxyl analogue hydroxychloroquine (HCQ) are commonly used as csDMARDs for the treatment of a variety of CIRDs, including SLE and RA [68]. CQ and HCQ are weak bases that exert anti-inflammatory properties by accumulating within cytoplasmic acidic organelles (lysosomes and endosomes), where they increase the pH and inhibit the activity of acidic pH-dependent endosomal/lysosomal proteases. The inhibition of lysosomal activity in antigen-presenting cells results in decreased toll-like receptor (TLR) signaling and reduced antigen processing and antigen presentation by major histocompatibility complex (MHC) class II molecules [69,70]. Such molecular events ultimately lead to a reduced production of proinflammatory cytokines (particularly IL-6 and TNF) by immune cells [71,72].

A growing body of evidence suggests that HCQ regulates glucose homeostasis by exerting antihyperglycemic properties in subjects with and without diabetes [73,74]. Indeed, several studies have shown that HCQ use was associated with a significantly reduced risk of incident diabetes in nondiabetic subjects with different CIRDs such as RA [30,75,76,77,78], SLE [79], SS [80], and psoriasis/PsA [77,78]. Furthermore, a few small, short-term randomized controlled trials have demonstrated that the addition of HCQ (at a dose ranging from 400 to 600 mg/day) to sulfonylureas or insulin therapy in T2D patients led to significant reductions in HbA1c values without increasing the risk of hypoglycemia [81,82,83]. Rekedal et al. [84] retrospectively examined medical records of patients with diabetes and concomitant rheumatic disease initiating either HCQ or MTX therapy, with at least one HbA1c measurement before and within 12 months after treatment initiation. The authors found that HCQ users had a significant 0.54% greater reduction in HbA1c values as compared with the HbA1c reduction observed among MTX users [84]. A recent meta-analysis of randomized controlled trials confirmed that HCQ treatment was associated with a significant reduction in fasting plasma glucose, 2-hour postprandial glucose, and HbA1c values [85].

Short-term, real-world, prospective observational studies and randomized trials conducted in India have shown that the use of HCQ (at a dose of 400 mg/day) as an add-on treatment in patients with uncontrolled T2D who were on a combination of two or more oral antihyperglycemic agents (including metformin, pioglitazone, sulfonylureas, dipeptidyl peptidase-4 inhibitors, sodium-glucose co-transporter-2 inhibitors, and alpha-glucosidase inhibitors) was well tolerated and resulted in a significant improvement of glucose control (assessed by fasting and postprandial blood glucose and HbA1c values) from baseline, without occurrence of severe hypoglycemic episodes [86,87,88]. Based on these preliminary findings, HCQ (400 mg/day) was approved in 2014 by the Drug Controller General of India as a third-line (add-on) antihyperglycemic agent for patients with inadequately controlled T2D despite lifestyle management and combination therapy with sulfonylurea plus metformin [89].

More recently, Chakravarti and Nag [90] conducted a double-blind, placebo-controlled, parallel group study to assess the safety and efficacy of HCQ as an add-on therapy in 304 inadequately controlled T2D patients who were on stable combination therapy with glimepiride and metformin. Participants were randomized to a placebo or HCQ 200 mg, 300 mg, or 400 mg once daily. HCQ therapy was associated with a significant reduction in HbA1c values from baseline to 12 weeks: −0.78%, −0.91%, and −1.2% for HCQ 200 mg, 300 mg, and 400 mg, respectively, versus −0.13% with the placebo. Significantly greater reductions in fasting plasma glucose, postprandial plasma glucose, and body weight were also observed with all HCQ doses as compared with the placebo. Hypoglycemia was reported only in the 300 mg (1.2%) and 400 mg (2.1%) HCQ groups. Overall, the safety and tolerability profile of HCQ was favorable, except for gastrointestinal disturbances which were more frequent with the HCQ dose of 400 mg [90]. Notwithstanding, a careful risk-benefit assessment of HCQ is always needed for a cautious use of this drug as an antihyperglycemic agent. In this regard, specific conditions or circumstances under which HCQ should be contraindicated or used with caution in the context of T2D (due to the increased risk of HCQ-related toxicity and severe hypoglycemia) include: pre-existing retinopathy and/or maculopathy; history or risk for macular edema; concomitant use of other oculotoxic agents; pre-existing cardiomyopathy or heart failure; pre-existing myopathy and/or neuropathy, glucose-6-phosphate dehydrogenase deficiency; diabetes complicated by hypoglycemia unawareness; repeated episodes of severe hypoglycemia and/or malnutrition [74].

Even though the exact mechanisms of action behind the glucose-lowering properties of HCQ are still not completely understood, preclinical and clinical studies have suggested that HCQ may exert multifaceted effects on glucose homeostasis, including: improvement of peripheral insulin sensitivity, reduced hepatic insulin clearance and insulin/insulin-receptor complex degradation in hepatocytes, improvement of insulin secretion, increased production of the anti-inflammatory and insulin-sensitizing adipokine adiponectin, reduction in systemic inflammation and inflammation-related insulin resistance in adipocytes and skeletal muscle cells [73,86,91].

Some of the glucose-lowering properties of HCQ were initially inferred from studies on the parent drug CQ, which has been shown to increase insulin levels in patients with T2D by promoting insulin secretion and inhibiting insulin degradation [92]. On the one hand, a recent study conducted by Toledo et al. [93] in nondiabetic insulin-resistant adults without rheumatic diseases demonstrated that HCQ administration for 13 weeks at a dose of 400 mg/day, as compared to placebo, significantly improved skeletal muscle insulin sensitivity (assessed by hyperinsulinemic-euglycemic clamp and stable-isotope tracer methods) and enhanced systemic glucose clearance. By contrast, HCQ did not affect hepatic insulin sensitivity and insulin clearance, but led to a significant reduction in circulating IL-6 levels along with a significant increase in adiponectin levels [93]. On the other hand, the putative effect of HCQ on endogenous insulin secretion may be mediated by a direct stimulatory effect of HCQ on beta cells (insulinotropic effect) and/or by indirect effects involving a decrease in pancreatic islet inflammation and/or a reduction in glucotoxicity- and lipotoxicity-induced beta cell dysfunction as a consequence of the improvement in metabolic control after HCQ treatment initiation. Thus, future mechanistic studies are warranted to clearly understand the exact mechanisms underlying the antihyperglycemic properties of HCQ in subjects with and without diabetes. Additionally, large randomized-controlled trials of long duration are needed to establish the long-term safety and efficacy profile of this drug in patients with CIRDs and comorbid T2D. However, it is also worth reminding that HCQ use has been associated with a reduced risk of overall cardiovascular events (including myocardial infarction, stroke, or venous thromboembolism) in patients with SLE or RA [94]. Cardiovascular benefits exerted by HCQ may rely on its lipid-lowering properties, inhibition of platelet aggregation, reduction in red blood cell aggregation, and on its anti-atherosclerotic and anti-thrombotic effects [95,96,97]. Yet, it is plausible that such benefits are also driven by the HCQ-mediated improvement of glucose homeostasis and insulin resistance [98], the latter being a well-established independent cardiovascular risk factor in a variety of populations, including the general population as well as patients with diabetes and CIRDs [5,77,99].

### 3.3. Sulfasalazine (SSZ)

Sulfasalazine (SSZ) is a csDMARD used for the treatment of ulcerative colitis and RA in children and adults. SSZ is a prodrug which is cleaved in vivo to the 5-aminosalicylic acid (mesalazine or mesalamine, a salicylate) and sulfapyridine (a sulfonamide antibiotic) [100]. Although the exact mechanisms of action of SSZ are not entirely clear, the anti-inflammatory and immunomodulatory properties of this drug appear to rely primarily on the SSZ ability to inhibit the activation of the transcription factor NF-kB, thus suppressing the transcription of NF-kB-responsive proinflammatory genes and cytokines, including TNF [100,101].

SSZ has previously been reported to lower HbA1c in a patient with SLE and T2D, who achieved normoglycemia after SSZ initiation [102]. However, other case reports and case series have attributed the HbA1c-lowering effect of SSZ to hematological changes rather than to possible glucose-lowering effects exerted by the drug [103,104]. The cause for the falsely low HbA1c values was mostly related to the SSZ-induced hemolysis. In line with a false lowering of HbA1c values, levels of blood glucose and fructosamine (an additional marker of medium-term glucose control, which is not affected by anemia, hemolysis, or variant hemoglobin) were not affected by SSZ therapy [103,104]. It has been suggested that even mild, subclinical hemolysis that does not cause anemia may have a significant impact on HbA1c values [103,105]. Hemolysis can lower HbA1c by shortening the lifespan of red blood cells: in fact, the period of time in which the hemoglobin is exposed to blood glucose shortens as red blood cell turnover increases, thus reducing the magnitude of hemoglobin glycation. Since the false lowering of HbA1c has also been observed with other sulphonamide drugs such as dapsone [103,104,106], sulphonamide pharmacophore has been proposed to contribute to such an effect. Indeed, a recent observational, population-based study conducted in T2D patients with an incident prescription of SSZ (received after the diagnosis of T2D) showed that SSZ initiation was associated with a significant 6-month reduction in HbA1c (mean reduction of −9 ± 16 mmol/mol, which is equivalent to −0.9 ± 1.4%) [107]. However, reduction in HbA1c values correlated with hematological changes consistent with hemolysis, namely a significant increase in mean cell volume and a significant decrease in red blood cell count [107]. These findings highlight a possible limitation of HbA1c as a reliable marker of glucose control in diabetic patients on SSZ therapy. Therefore, studies using continuous glucose monitoring or alternative markers of glucose control other than HbA1c are warranted to clearly establish the true impact of SSZ on glucose homeostasis in CIRD patients with and without T2D.

### 3.4. Leflunomide (LEF)

Leflunomide (LEF) is a csDMARD used for the treatment of RA and PsA. This drug exerts anti-inflammatory and immunomodulatory properties by inhibiting the mitochondrial enzyme dihydroorotate dehydrogenase (DHODH), which plays a crucial role in the de novo synthesis of the pyrimidine ribonucleotide uridine monophosphate (rUMP). As such, leflunomide prevents rUMP synthesis and reduces the proliferation of activated and autoreactive lymphocytes [108,109].

The active metabolite of leflunomide (also known as A77 1726) has been shown to directly inhibit the in vitro activity of p70 S6 kinase, which is a serine/threonine kinase that phosphorylates the insulin receptor substrate-1 (IRS-1) at serine 1101 and subsequently desensitizes insulin receptor signaling [110]. Chen et al. [111] showed that A77 1726 decreased IRS-1 phosphorylation at serine 1001 in 3T3-L1 adipocytes, C2C12 cells (a murine myoblast cell line), and L6 cells (a rat myoblast cell line). A77 1726 also enhanced insulin-stimulated glucose uptake in 3T3-L1 adipocytes and L6 myotubes, and amplified the insulin-stimulated translocation of glucose transporter type 4 (GLUT4) to the plasma membrane of L6 myotubes. Additionally, leflunomide treatment normalized blood glucose levels and overcame insulin resistance in glucose and insulin tolerance tests performed in ob/ob and high-fat diet (HFD)-fed mice [111]. Although these findings need to be validated in large human studies, they suggest LEF as a potential insulin-sensitizing agent for treatment of patients with both CIRDs (particularly RA and PsA) and T2D.

## 4. Targeted Synthetic DMARDs (tsDMARDs)

The family of targeted synthetic DMARDs (tsDMARDs) comprises phosphodiesterase 4 (PDE4) inhibitors and JAKIs, which have been shown to exert potential antihyperglycemic properties in some studies.

### 4.1. Phosphodiesterase 4 (PDE4) Inhibitors

Apremilast is an orally administered small molecule which acts as a selective inhibitor of PDE4 and has been approved by the FDA for treatment of active PsA and moderate-to-severe plaque psoriasis [112]. The enzyme PDE4 regulates intracellular levels of cyclic adenosine monophosphate (cAMP) and, thus, the expression of effector and regulatory cytokines involved in immune and inflammatory processes [113]. Apremilast exerts an anti-inflammatory activity through the inhibition of PDE4 and subsequent cAMP degradation, which results in decreased production of several proinflammatory cytokines and chemokines—including interferon (IFN)-γ, TNF, IL-12, IL-23, C-X-C motif chemokine ligand 9 (CXCL9), and CXCL10—accompanied by an increased production of anti-inflammatory cytokines such as IL-10 [113,114,115]. Short-term studies and case reports have documented significant reductions in fasting plasma glucose, HbA1c values, and body weight after apremilast treatment in diabetic patients with psoriasis and PsA [116,117,118,119].

A proof of concept for the potential role of pharmacological PDE4 inhibition on glucose homeostasis comes from a 12-week, randomized, double-blind, placebo-controlled multicenter study on roflumilast, a PDE4 inhibitor used for treatment of severe chronic obstructive pulmonary disease (COPD). In this study, authors found a significantly greater reduction in HbA1c (least square mean of −0.45%) and fasting plasma glucose levels, accompanied by a significant increase in C-peptide levels in patients with newly diagnosed T2D without COPD [120]. These findings suggest that PDE4 inhibition may improve beta cell function. Accordingly, PDE4C and PDE4D are expressed in pancreatic beta cells and are thought to control insulin secretion [121,122]. Moreover, selective inhibition of PDE4 has been shown to prevent diabetes in non-obese diabetic (NOD) mice [122]. In addition, roflumilast has been shown to improve peripheral insulin sensitivity in adults with prediabetes and overweight/obesity [123], suggesting that PDE4 may participate in the pathophysiology of peripheral insulin resistance through direct and/or indirect effects. A similar concept has been proposed for other PDE types such as PDE5 [124]. Therefore, large prospective studies are needed to assess whether PDE4 inhibitors exert antihyperglycemic properties in the long-term period.

### 4.2. Janus Kinase (JAK) Inhibitors (JAKIs)

JAK inhibitors (JAKIs) are the most studied kinase inhibitors in CIRDs. This category of tsDMARDs includes tofacitinib and baricitinib, which are FDA-approved drugs for the treatment of patients with moderately to severely active RA who had an inadequate response to other antirheumatic therapies [125,126]. The JAK/signal transducer and activator of transcription (STAT) pathway is crucial for transducing signals from a series of metabolically relevant hormones and cytokines such as growth hormone, leptin, IL-4, IL-6, and IFN-γ. A growing body of evidence suggests that this pathway is dysregulated in metabolic diseases, including obesity and T2D [127]. A study conducted in streptozotocin-induced diabetic rats showed that JAK-STAT inhibition with tofacitinib significantly reduced TNF, IL-6, and serum amyloid A (SAA) levels as compared to diabetic untreated rats [128]. These changes were accompanied by a significant improvement in insulin secretion and insulin sensitivity when tofacitinib was administered in combination with aspirin (a well-known inhibitor of the NF-kB pathway), suggesting that simultaneous inhibition of JAK-STAT and NF-κB signaling pathways may mitigate insulin resistance and hyperglycemia in T2D [128]. Collotta et al. [129] investigated the effects of the JAK1/JAK2 inhibitor baricitinib in a murine high-fat-high-sugar diet model. The authors found that baricitinib treatment restored insulin signaling in the liver and skeletal muscle, and led to improvements in diet-induced myosteatosis, mesangial expansion, and proteinuria. The skeletal muscle and renal protection were due to the local inhibition of JAK2-STAT2 pathway mediated by baricitinib. Furthermore, baricitinib administration was associated with a significant reduction in the circulating levels of proinflammatory cytokines that signaled through the JAK-STAT pathway, such as TNF, IL-1β, and IFN-γ. Based on these findings, the authors suggested that the JAK2-STAT2 pathway may represent a valid candidate for the treatment of metabolic diseases, with JAKIs having the potential to be repurposed for treatment of T2D and its complications [129]. In this regard, it is worth noting that the JAK-STAT pathway represents one of the major pathways that transduce inflammatory signals and it also contributes to diabetic nephropathy [130]. A phase 2, randomized, placebo-controlled, double-blind, dose-ranging study conducted in patients with T2D at high risk for progressive diabetic kidney disease showed that baricitinib (administered orally at a dose of 4 mg/day), as compared with a placebo, led to a significant 41% reduction in albuminuria (morning urine albumin/creatinine ratio) [131]. Moreover, different inflammatory biomarkers (urinary CXCL10 and C-C motif ligand 2, plasma soluble TNF receptors 1 and 2, SAA, intercellular adhesion molecule 1, and vascular cell adhesion molecule 1) showed dose-dependent decreases from baseline with baricitinib treatment [131]. Future prospective studies are warranted to clarify whether different types and doses of JAKIs exert a beneficial role in terms of management of glucose control and chronic diabetes complications among patients with CIRDs and T2D.

## 5. Biologic DMARDs (bDMARDs)

### 5.1. TNF Inhibitors

TNF-α is a proinflammatory cytokine produced by immune cells such as monocytes and macrophages in response to inflammatory processes [132]. In addition, TNF-α plays a central role in the pathogenesis of insulin resistance [133], T2D [134], and various CIRDs such as RA, PsA, and AS [135,136,137]. TNF inhibitors are FDA-approved drugs used for treatment of different CIRDs such as RA, AS, PsA, juvenile idiopathic arthritis, plaque psoriasis, and polyarticular juvenile idiopathic arthritis [138]. For example, TNF inhibitors are widely used for treatment of patients with RA to counterbalance the excessive TNF levels, which account for joint inflammation and destruction of joint cartilage and bone [139]. The currently available TNF inhibitors include etanercept, adalimumab, infliximab, golimumab, and certolizumab pegol [138].

Several studies have investigated the interaction between TNF-α and the insulin signaling pathway. TNF-α promotes insulin resistance by reducing insulin-mediated tyrosine phosphorylation of the insulin receptor (IR) and by inhibiting the tyrosine kinase activity of IR via serine phosphorylation of IRS-1 [133,140]; it also inhibits the activity of phosphatidylinositol 3-kinase (PI-3 kinase) [141], the autophosphorylation of IR, and the subsequent translocation of GLUT4 to the plasma membrane [142]. These molecular events may partly explain the frequent associations among CIRDs (e.g., RA and PsA) and T2D [22,27]. Hence, it has been postulated that modulation of the TNF signaling pathway may improve insulin sensitivity in patients with CIRDs with or without T2D. In this regard, several studies have shown that anti-TNF therapy (based on the administration of infliximab, etanercept, or adalimumab) was able to reduce insulin resistance—measured by the HOMA-IR—in nondiabetic patients with RA [21,143,144,145]. Infliximab (a chimeric monoclonal, high affinity antibody which binds to and neutralizes the soluble and transmembrane forms of TNF-α) has been shown to ameliorate TNF-induced insulin resistance in 3T3-L1 adipocytes in vitro by restoring the insulin signaling pathway via mitigation of protein tyrosine phosphatase 1B (PTP1B) activation [146]. Another study suggested that infliximab may have beneficial effects on insulin sensitivity in RA and AS patients with a high degree of insulin resistance at baseline [147]. However, this study did not compare anti-TNF therapy with other DMARDs [147].

In a large retrospective cohort study conducted in patients with a diagnosis of either RA or psoriasis, the use of a TNF inhibitor (infliximab, etanercept, or adalimumab) was associated with a reduced risk of developing diabetes as compared with initiation of other nonbiologic DMARDs [77]. Another retrospective study conducted in patients with RA or Crohn’s disease and T2D who received anti-TNF agents (infliximab or etanercept) in larger therapeutic doses for up to 10 years found that initiation of anti-TNF treatment was associated with a significant improvement in fasting blood glucose, HbA1c (on average −1.0%), and triglyceride values [148]. In a 6-month randomized, placebo-controlled trial conducted in obese subjects with abnormal glucose homeostasis and marked subclinical inflammation, etanercept (a fully human recombinant dimeric fusion protein consisting of two extracellular ligand-binding domains of the TNF p75 receptor linked to the Fc portion of human immunoglobulin G1) led to a significant improvement in fasting blood glucose [149]. The improvement in fasting blood glucose was also accompanied by an increase in the ratio of high-molecular weight adiponectin (HMWA, the most biologically active form of adiponectin) to total adiponectin, and by a reduction in soluble intercellular adhesion molecule 1 (sICAM-1) levels [149]. However, other short-term studies have shown a lack of effect of etanercept on markers of insulin secretion and insulin sensitivity in patients with CIRDs and T2D or metabolic syndrome [150,151,152]. Long-term prospective studies are required to clarify the existence of clinical benefits (in terms of glucose control) deriving from TNF antagonism in patients with CIRDs and T2D.

### 5.2. IL-1 Blocking Therapies

The IL-1 family includes both proinflammatory and anti-inflammatory cytokines. The balance between proinflammatory and anti-inflammatory members of the IL-1 family affects the degree of inflammation and the severity of many CIRDs. Among the proinflammatory family members, IL-1α is primarily associated with skin diseases, whereas IL-1β has emerged as a pivotal contributor to inflammation in autoinflammatory diseases and its expression is increased within the articular environment during arthritis [153]. IL-1 blocking agents are used for the treatment of different rheumatic diseases (including RA and gout) and autoinflammatory disorders such as cryopyrin-associated periodic syndrome, adult-onset Still’s disease, systemic-onset juvenile idiopathic arthritis, and familial Mediterranean fever [154,155].

The availability of IL-1-targeting agents unveiled the pathophysiological role of IL-1-mediated inflammation in a wide range of disorders beyond the spectrum of CIRDs, including T2D and CVD which are frequent comorbidities in rheumatic patients [154]. In T2D, chronic hyperglycemia exerts toxic effect on beta cells (glucotoxicity) and leads to beta cell dysfunction, impaired insulin secretion, and beta cell apoptosis partly by inducing inflammatory processes. Although IL-1β is not expressed in normal human pancreatic islets [156], Maedler et al. [157] showed that the IL-1β/NF-kB pathway mediated, at least in part, the deleterious effects of hyperglycemia on human beta cells. The authors found that in vitro exposure of pancreatic islets from nondiabetic organ donors to high glucose levels led to an increased production and release of IL-1β, accompanied by NF-kB activation, Fas upregulation, DNA fragmentation, and beta cell dysfunction and apoptosis. Of note, IL-1 receptor antagonism protected against such deleterious effects exerted by hyperglycemia on pancreatic beta cells. Importantly, beta cells were also identified as the cellular source of glucose-induced IL-1β within the pancreatic islets. In fact, double immunostaining of pancreatic sections for IL-1β and insulin revealed beta cell expression of IL-1β in sections of pancreas from poorly controlled T2D patients but not in nondiabetic control subjects [157]. IL-1β was also induced in most beta cells of severely hyperglycemic diabetes-prone gerbils *Psammomys obesus* fed a high-energy diet which barely expressed insulin. Treatment of these animals with phlorizin (a natural glucoside that prevents renal glucose reabsorption via inhibition of sodium-glucose co-transporters 1 and 2, thus inducing renal glycosuria) normalized plasma glucose, restored insulin stores, and prevented IL-1β expression within the pancreatic beta cells [157]. Overall, these findings suggest an involvement of inflammation in the pathogenesis of glucotoxicity and highlight the role of the IL-1β/NF-kB pathway as a promising target to preserve or restore beta cell mass and function in patients with T2D.

An increase in intra-islet IL-1β concentrations following hyperglycemia may depend on the activation of the NLRP3 inflammasome in pancreatic beta cells and islet-infiltrating immune cells (especially macrophages), resulting in the processing of pro-IL-1β to the biologically active IL-1β [7,158]. In turn, IL-1β released from beta cells promotes the recruitment and activation of macrophages, which release additional amounts of IL-1β within the pancreatic islet microenvironment, thereby triggering beta cell apoptosis, inhibiting insulin secretion, and causing further increases in blood glucose levels through a vicious cycle. IL-1 receptor blockade inhibits IL-1β signaling in beta cells and macrophages, thus breaking this vicious cycle and restoring the beta cell insulin secretory capacity. In this view, T2D can be considered to be a “waxing and waning” islet inflammatory disease characterized by flares induced by hyperglycemia, as it has been suggested by Donath and colleagues [7]. Therefore, short-term IL-1 receptor blockade may represent an effective remission-induction therapy following metabolic relapses in diabetic patients with CIRDs [7].

Apart from hyperglycemia, other stimuli may promote intra-islet IL-1β expression and accumulation. Islet amyloid polypeptide (IAPP, also known as amylin) is co-secreted with insulin from beta cells; in T2D, IAPP tends to aggregate and to form amyloid fibrils that are toxic to beta cells and contribute to beta cell loss via inducing apoptosis [159,160]. It has been shown that IAPP oligomers could trigger the NLRP3 inflammasome, thus generating mature IL-1β in murine bone marrow-derived macrophages [161]. Accordingly, mice transgenic for human IAPP exhibited marked regions within the pancreatic islet that did not stain for insulin, whereas they displayed evidence of IL-1β staining that co-localized with amyloid and macrophages [161]. In a study conducted by Park et al. [162], human IAPP aggregates were found to increase IL-1β levels in cultured human islets, which correlated with beta cell Fas upregulation, caspase-8 activation, and apoptosis. All these events were reduced by treatment with anakinra, a recombinant human IL-1 receptor antagonist which prevents the activity of IL-1α and IL-1β by binding to the IL-1 receptor. Anakinra also improved culture-induced beta cell dysfunction and restored impaired proIAPP processing, resulting in lower amyloid formation [162]. IL-1β treatment enhanced the impaired proIAPP processing and increased amyloid formation in cultured human and human IAPP-expressing mouse islets, whereas IL-1 receptor antagonism prevented such events [162]. These results suggest the existence of another vicious cycle accounting for IL-1β-mediated beta cell loss, which involves IL-1β as well as the generation of IAPP aggregates.

Free fatty acids (FFAs) can also induce IL-1β and other proinflammatory cytokines (such as IL-6 and IL-8) in human islets [163]. In turn, elevated glucose concentrations further enhance FFA-induced expression of proinflammatory factors in human islets [163]. In this regard, FFA-induced lipotoxicity in pancreatic beta cells may be mediated, at least partly, by local increases in IL-1β concentrations. Hence, targeting lipotoxicity may represent an adjuvant therapeutic approach to prevent or reverse IL-1β-mediated beta cell dysfunction in patients with CIRDs and comorbid T2D.

Larsen et al. [164] first conducted a double-blind, parallel-group trial involving 70 patients with T2D who were randomly assigned to receive a placebo or 100 mg of anakinra subcutaneously once daily for 13 weeks. At 13 weeks, the anakinra group, as compared with the placebo group, showed a significantly lower HbA1c value (−0.46%), an enhanced insulin secretion (as assessed by stimulated C-peptide levels in response to a 2-h oral glucose tolerance test), and a significant reduction in proinsulin-to-insulin ratio [164], the latter being a marker of beta cell dysfunction and reduced insulin secretory capacity [165,166]. These changes were accompanied by a significant reduction in CRP and IL-6, suggesting that IL-1 receptor blockade with anakinra may improve glucose control and beta cell secretory function even by decreasing the production of additional markers of systemic inflammation [164].

A randomized, double-blind, crossover study conducted on nondiabetic, obese subjects with metabolic syndrome showed that anakinra (administered at a dose of 150 mg subcutaneously once daily), as compared with a placebo, led to a significantly lower degree of inflammation (as evidenced by a reduction in circulating CRP levels and leukocyte numbers) accompanied by a significant increase in the disposition index, which reflected an improvement in beta cell function [167]. Nevertheless, anakinra did not significantly improve insulin sensitivity (as measured by euglycemic hyperinsulinemic clamp) as compared with a placebo [167]. In line with these findings, a double-blind, randomized, placebo-controlled crossover study conducted on subjects with impaired glucose tolerance demonstrated that anakinra (administered at a dose of 150 mg/day for 4 weeks) was able to significantly improve the first-phase insulin secretion as compared with a placebo [168]. These results provide further evidence for the involvement of IL-1β in the progressive decline in insulin secretion associated with T2D.

Interestingly, some studies have demonstrated antihyperglycemic properties exerted by other IL-1 blocking therapies. For instance, a 4-month randomized, double-blind, placebo-controlled study conducted on 551 metformin-treated patients with T2D was designed to assess the effects on HbA1c and the safety profile of different monthly doses of canakinumab, a human monoclonal antibody targeting IL-1β [169]. Canakinumab treatment was safe and well tolerated, and led to a numerical HbA1c reduction (between 0.19% and 0.31%) [169]. Similarly, Cavelti-Weder et al. [170] evaluated the safety and biological activity of the human monoclonal anti-IL-1β antibody gevokizumab in a placebo-controlled, dose-escalation study involving a total of 98 patients with T2D. Gevokizumab treatment was safe and led to a significant reduction in HbA1c values (−0.85%) after 3 months, which was accompanied by augmented C-peptide secretion, increased insulin sensitivity, and decreased CRP levels [170]. Finally, a meta-analysis of 2921 individuals from eight phase I-IV studies demonstrated a significant overall HbA1c-lowering effect of IL-1 antagonism [171].

#### 5.2.1. IL-1 Receptor Blockade in RA and T2D

RA is a chronic inflammatory, autoimmune disease characterized by the presence of synovial inflammation (synovitis) that eventually leads to the destruction of joint cartilage and bone, with resulting joint swelling, pain, and disability. T2D is a common comorbidity in patients with RA [22,28], and a growing body of evidence suggests that IL-1β is a shared proinflammatory mediator in RA and T2D [172]. In RA, excessive amounts of IL-1β produced by tissue macrophages contribute to cartilage destruction, inhibition of new matrix synthesis, bone loss, and joint destruction via the modulation of synovial leukocyte infiltration and pannus organization [173,174]. As we previously mentioned, IL-1β contributes to beta cell dysfunction and apoptosis induced by hyperglycemia and other metabolic stressors in T2D [172]. The IL-1β pathway has also been suggested to drive systemic and tissue inflammation in T2D, thus contributing to peripheral insulin resistance and T2D-related cardiovascular complications [175].

IL-1 blocking agents (particularly anakinra) are used for the treatment of RA, and some studies have shown the efficacy of IL-1 receptor antagonism in patients with RA and comorbid T2D [172]. Of note, a multicenter, randomized, open-label, prospective, controlled, parallel-group study, called TRACK (The Treatment of Rheumatoid Arthritis and Comorbidities with Kineret), investigated whether IL-1 inhibition with anakinra, as compared with TNF inhibitors (e.g., etanercept, adalimumab, infliximab, certolizumab pegol, or golimumab), could lead to improvements in both metabolic and inflammatory parameters in patients with RA and T2D [176]. In total, 41 participants with RA and T2D were randomized, and 39 eligible participants were treated. In a linear mixed model adjusted for relevant RA and T2D clinical confounders (age, male sex, anti-cyclic citrullinated peptide antibody positivity, use of corticosteroids, RA duration, T2D duration, use of oral antidiabetic medications, and body mass index), anakinra treatment was associated with a significant reduction in HbA1c values (expressed as percentage) after 3 months (β −1.04, *p* < 0.001, and 95% CI −1.52 to −0.55) and after 6 months (β −1.24, *p* < 0.001, and 95% CI −1.75 to −0.72), whereas participants in the TNF inhibitor group showed a slight (non-significant) decrease in HbA1c values (β −0.17 at 3 months; β −0.06 at 6 months). With regard to RA, both anakinra and TNF inhibitor groups showed a significant decrease in disease activity. Interestingly, the authors found a significant correlation between decreasing levels of HbA1c and a reduction in disease activity (assessed by the disease activity score in 28 joints), suggesting that the inflammatory pathogenic mechanisms of T2D may be more pronounced in the context of RA. No severe adverse events (including hypoglycemic episodes) were observed during the entire duration of the study [176].

Although the TRACK study was prematurely stopped due to early benefit, the authors continued to monitor the study participants [177]. After a mean follow-up of 18 months, RA clinical response was maintained in both anakinra and TNF inhibitor groups. The glucocorticoid dose was reduced in both groups, but a significantly greater proportion of anakinra-treated participants discontinued such drugs as compared with TNF inhibitor-treated subjects (53.3% vs. 28.6%, *p* = 0.004). Although no significant difference was observed between the anakinra and TNF inhibitor groups in terms of HbA1c values, a significantly greater proportion of anakinra-treated participants required antidiabetic drug regimen adjustments (consisting of drug dose reduction, switch from combination therapy to monotherapy, or drug discontinuation) as compared with participants treated with TNF inhibitors (53.3% vs. 7.1%, *p* = 0.008) [177].

In a 6-month longitudinal cohort study that consecutively recruited patients with RA and comorbid T2D receiving anakinra (100 mg/day) or TNF inhibitors, anakinra-treated patients exhibited a significant improvement in both beta cell function and insulin resistance (as assessed by HOMA2-%β and HOMA2-IR, respectively), as well as a significant reduction in plasma glucagon levels [178]. Conversely, patients treated with TNF inhibitors did not show any significant difference in these metabolic parameters [178]. These findings suggest that pharmacological blockade of IL-1 receptor may exert additional antihyperglycemic properties beyond its main mechanism of action on beta cells, such as improvement of inflammation-driven insulin resistance and inhibition of excessive glucagon secretion from pancreatic alpha cells. Indeed, hyperglucagonemia represents an important contributor to the pathophysiology of T2D [179].

Therefore, IL-1 receptor antagonism with anakinra may represent a tailored therapy for patients with RA and comorbid T2D. Anakinra could offer various advantages in this subset of patients, including: (i) improvement in joint involvement and RA disease activity or induction of disease remission by reducing tissue and systemic inflammation; (ii) discontinuation of corticosteroid therapy or reduction in the daily corticosteroid dose (“steroid-sparing effect”) and glucocorticoid-mediated diabetogenic effects, resulting in prevention or mitigation of steroid-induced diabetes; (iii) improvement in beta cell function and glucose control, which is primarily mediated by the protective effects of anakinra against IL-1β-induced beta cell dysfunction and apoptosis. Given that the antidiabetic action of anakinra mainly relies on the preservation of beta cell mass and function, this drug may be a good therapeutic option to counteract IL-1β-driven beta cell dysfunction and to halt or slow down the progressive decline in insulin secretion occurring over time in RA patients with comorbid T2D and insulin resistance. However, larger studies are needed to establish the safety and efficacy profile of IL-1 blocking therapies in patients with RA and T2D in the long term. Furthermore, studies involving RA patients without diabetes could elucidate whether IL-1 antagonism may have an impact on the prevention of prediabetes and T2D in selected at-risk groups (e.g., patients with RA and obesity and/or metabolic syndrome, or patients with RA on high-dose corticosteroid therapy).

#### 5.2.2. IL-1β Pathway and CVD

IL-1β may contribute to cardiovascular morbidity and mortality in patients with and without T2D [175,180]. A large randomized, double-blind, placebo-controlled trial called the Canakinumab Anti-Inflammatory Thrombosis Outcome Study (CANTOS) involving 10,061 patients with previous myocardial infarction assessed whether canakinumab could prevent recurrent cardiovascular events in subjects with a persistent proinflammatory response, defined as a high-sensitivity CRP level ≥ 2 mg/L. The primary efficacy end point was nonfatal stroke, nonfatal myocardial infarction, or cardiovascular death. The authors showed that canakinumab administered subcutaneously at a dose of 150 mg every 3 months resulted in a significant reduction in CRP level accompanied by a significantly lower rate of recurrent cardiovascular events as compared with a placebo (median follow-up 3.7 years) [181]. Canakinumab did not reduce lipid levels from baseline, suggesting that the reduction in major cardiovascular events was mediated by the anti-inflammatory therapy targeting the IL-1β innate immunity pathway [181]. A prespecified exploratory analysis of the CANTOS study found that canakinumab was also related to a dose-dependent reduction in hospitalization for heart failure (HHF) and in the composite of HHF or heart failure-related mortality as compared with a placebo [182]. This finding has important clinical implications for patients with T2D, since they are at increased risk for heart failure-related morbidity and mortality partly as a consequence of systemic inflammation [183]. Nevertheless, in the CANTOS study, canakinumab treatment was also associated with a significantly higher incidence of fatal infection as compared with a placebo, and patients who died from infection tended to be older and more likely to have diabetes than those who did not die from infection [181]. Moreover, IL-1β inhibition with canakinumab did not reduce incident diabetes among CANTOS participants over a median period of 3.7 years [184]. However, subjects with prediabetes and diabetes randomized to canakinumab demonstrated modest - but statistically significant - reductions in HbA1c values (between −0.1% and −0.3%) during the first 6 to 9 months, an effect that attenuated over time, probably due to the study design that allowed lifestyle interventions and alterations in other antidiabetic medications [184].

The results from the CANTOS study served as a proof of concept for the ability of canakinumab to reduce cardiovascular mortality by virtue of its marked anti-inflammatory properties, making IL-1β antagonism an attractive therapeutic approach to reduce cardiovascular risk and potentially improve glucose homeostasis in patients with and without T2D. Yet, the potential risk for fatal infection related to canakinumab may undoubtedly represent a strong limitation to future clinical applications that needs to be more extensively investigated for a thorough risk-benefit analysis of this drug, particularly in the context of T2D.

### 5.3. IL-6 Inhibitors

Excessive chronic IL-6 production promotes the development of insulin resistance and T2D by impairing the phosphorylation of IR and IRS-1 via the upregulation of SOCS-3 (suppressor of cytokine signaling 3) [185], a potential inhibitor of insulin signaling [186]. Little evidence for a possible antihyperglycemic role of IL-6-targeted therapy comes from tocilizumab (TCZ), a recombinant humanized anti-IL-6 receptor monoclonal antibody approved for treatment of patients with moderate-to-severe RA. Ogata et al. [187] first reported that TCZ initiation led to a significant reduction in HbA1c values after 1 and 6 months of TCZ treatment (−0.8% and −1.2%, respectively) in diabetic patients with RA. The daily prednisolone dosage was tapered in all patients 6 months after the initiation of TCZ treatment [187].

A retrospective study conducted in RA patients with and without diabetes revealed that TNF inhibitors and TCZ led to significantly lower HbA1c values at 1 month and 3 months after treatment initiation, with significant changes in HbA1c values persisting in each group regardless of the presence of diabetes [188]. However, the 3-month treatment HbA1c values were significantly lower (TNF inhibitors 6.1% and TCZ 5.8%, *p* = 0.010) and the changes in HbA1c (ΔHbA1c) were significantly greater in the TCZ group (TNF inhibitors 0.1% and TCZ 0.4%, *p* < 0.001), suggesting that TCZ decreases HbA1c levels in RA patients to a greater extent than TNF inhibitors [188].

It is also worth noting that the antihyperglycemic properties of IL-1 blocking therapies may partly be mediated by the ability of these agents to concomitantly reduce IL-6 levels. IL-1β is a potent inducer of IL-6 in several cell types such as fibroblasts, smooth muscle cells, endothelial cells, and monocytes [189,190,191,192]. In turn, IL-6 elicits the acute phase response by stimulating hepatocytes to synthesize acute-phase proteins, including CRP [193,194]. In line with this hypothesis, 13-week anakinra treatment has been shown to significantly reduce HbA1c values as well as IL-6 and CRP levels in patients with T2D [164]. Similar remarks can apply to HCQ, which has been shown to improve skeletal muscle insulin sensitivity, enhance systemic glucose clearance, and concomitantly reduce circulating IL-6 levels in insulin-resistant adults [93]. Therefore, further studies are warranted to better elucidate the role of IL-6 and IL-6 inhibitors on glucose homeostasis in CIRD patients with and without T2D.

### 5.4. Abatacept

Abatacept (CTLA4-Ig) is a bDMARD approved for use in patients with highly active and progressive RA who were not previously treated with MTX, or for treatment of moderate-to-severe active RA in patients with an inadequate response to previous therapy with at least one cDMARD [195]. Abatacept is designed to interfere with T-cell co-stimulation mediated by the CD28-CD80/86 pathway: it is a fusion protein including the extracellular domain of the cytotoxic T lymphocyte-associated antigen-4 (CTLA-4) combined with the Fc domain of human immunoglobulin G1 (IgG1). The abatacept fragment comprising the extracellular domain of CTLA4 binds to CD80/CD86 receptors on the surface of antigen-presenting cells, preventing or displacing the binding of CD80/CD86 to CD28 receptor expressed on the surface of T cells, thereby inhibiting T-cell activation/proliferation and B-cell immunological response [196].

Ursini et al. [197] first reported the case of a 47-year-old nondiabetic obese woman with RA who showed a remarkable improvement in insulin resistance (determined by HOMA-IR) 4 weeks after abatacept initiation. Thereafter, a study published by the same group showed that nondiabetic patients with RA exhibited a significant increase in the insulin sensitivity index (calculated through the equation proposed by Matsuda, which provides a reliable estimation of the whole-body insulin sensitivity), along with a significant, although modest, reduction in HbA1c values (−0.2%) and glucose and insulin area under the curves during the oral glucose tolerance test at 6 months after abatacept initiation [198]. Nevertheless, no significant differences were observed in markers of beta cell function (insulinogenic index and oral disposition index) [198]. These findings suggest that treatment with abatacept may improve whole-body insulin sensitivity in RA patients without affecting beta cell function. Ozen et al. [30] studied patients with RA and ≥1 year participation in a U.S.-wide longitudinal observational cohort (the National Data Bank for Rheumatic Diseases) without baseline diabetes from 2000 through 2014. Remarkably, authors found that abatacept was associated with a significantly reduced risk of incident diabetes in this cohort [30].

Although the exact mechanisms behind the effects of abatacept on insulin sensitivity and glucose homeostasis are still unclear, preclinical evidence supports the hypothesis of a relevant role played by both macrophages and T cells in the initiation of adipose tissue inflammation and subsequent insulin resistance [199]. It has been shown that T-cell infiltration may even precede the macrophage accumulation within murine and human visceral adipose tissue derived from mouse models of obesity-mediated insulin resistance and from patients with T2D, respectively [200,201]. Therefore, T-cell targeted immunotherapies may represent a valid therapeutic tool to indirectly improve insulin resistance by reducing the activation of adipose tissue-infiltrating T cells. However, mechanistic studies are required to confirm this hypothesis.

## 6. Discussion

Systemic inflammation represents a shared pathophysiological mechanism which underlies the frequent clinical associations among CIRDs and insulin resistance, T2D, and chronic diabetes complications. Targeted anti-inflammatory therapies have long been suggested for both prevention and treatment of T2D [202]. Therefore, therapeutic approaches targeting inflammation represent highly desirable interventions to improve both rheumatic disease activity and glucose control in patients with CIRDs and comorbid T2D. Targeted anti-inflammatory therapies may also represent a valid tool to prevent the development of prediabetes and diabetes in rheumatic patients, particularly in those with traditional risk factors and on long-term high-dose corticosteroid therapy. Controlling inflammation in CIRDs may provide additional clinical benefits, including prevention of chronic diabetes complications, reduction in cardiovascular morbidity and mortality, improvement of quality of life and medication adherence, as well as reduction in healthcare costs related to disease relapses and hospitalizations. The use of DMARDs in CIRD patients can also be advantageous in reducing the daily corticosteroid dose (steroid-sparing effect) [203], thereby mitigating the risk of incident diabetes (including steroid-induced diabetes, which is highly prevalent in these patients) [204,205] or preventing poor glucose control in diabetic subjects. Indeed, prolonged corticosteroid exposure deriving from long-term corticosteroid therapy leads to diabetogenic effects by enhancing hepatic endogenous glucose production and inducing insulin resistance in all insulin-sensitive tissues [206]. Furthermore, long-term corticosteroid therapy can independently contribute to increased cardiovascular risk, osteoporosis, and fragility fractures [207]. In view of the above, it is clear that minimizing corticosteroid exposure represents a key strategy to improve long-term outcomes in patients with CIRDs [207]. In this regard, the 2019 European League Against Rheumatism (EULAR) recommendations for the management of RA outline the importance of using glucocorticoids as a bridging therapy until csDMARDs show their efficacy, although specifying that glucocorticoids should be tapered as rapidly as clinically feasible (aiming at discontinuation within about 3 months) [208].

Additionally, the use of a DMARD to concomitantly target rheumatic disease activity and T2D could be advantageous in reducing polypharmacy in patients with CIRDs and comorbid diabetes.

**Figure 1:**
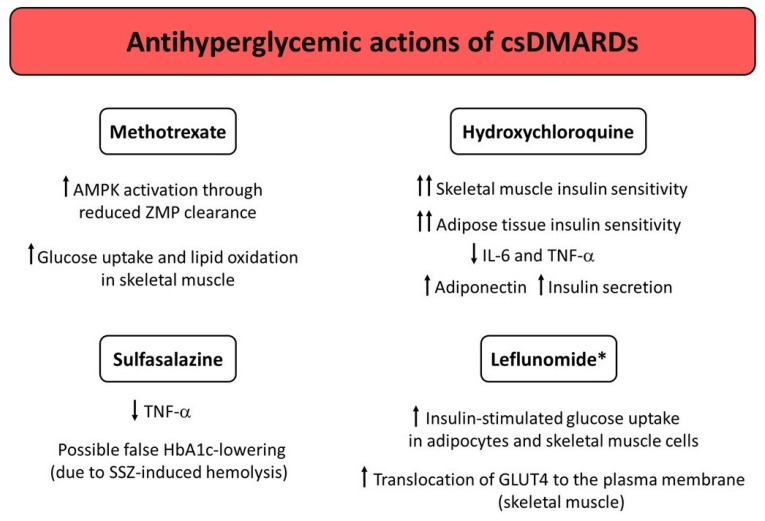
Potential mechanisms underlying the antihyperglycemic actions exerted by conventional synthetic DMARDs (csDMARDs). * Studies conducted in mice only. Abbreviations: AMPK, adenosine monophosphate-activated protein kinase; DMARDs, disease-modifying antirheumatic drugs; GLUT4, glucose transporter type 4; HbA1c, glycated hemoglobin; IL, interleukin; SSZ, sulfasalazine; TNF-α, tumor necrosis factor α; ZMP, aminoimidazole carboxamide ribonucleotide (AICAR) monophosphate.

**Figure 2:**
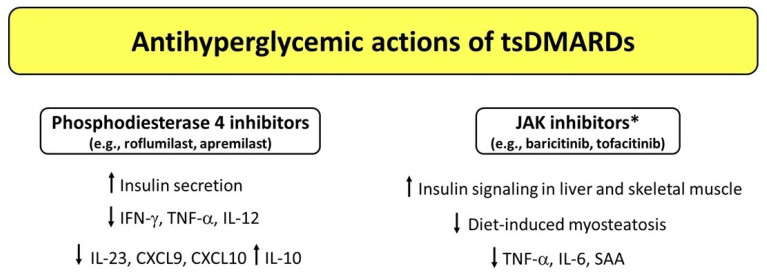
Potential mechanisms underlying the antihyperglycemic actions exerted by targeted synthetic DMARDs (tsDMARDs). * Studies conducted in mice (baricitinib) and rats (tofacitinib). Abbreviations: CXCL9, C-X-C motif chemokine ligand 9; CXCL10, C-X-C motif chemokine ligand 10; DMARDs, disease-modifying antirheumatic drugs; IFN-γ, interferon-γ; IL, interleukin; SAA, serum amyloid A; TNF-α, tumor necrosis factor α.

**Figure 3:**
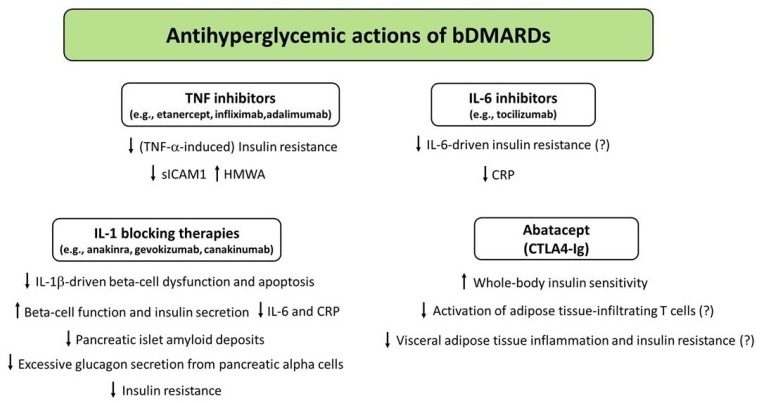
Potential mechanisms underlying the antihyperglycemic actions exerted by biologic DMARDs (bDMARDs). Abbreviations: CRP, C-reactive protein; DMARDs, disease-modifying antirheumatic drugs; HMWA, high-molecular weight adiponectin; IL, interleukin; sICAM-1, soluble intercellular adhesion molecule-1; TNF-α, tumor necrosis factor α.

Over the last few years, a growing body of evidence has shown that different DMARDs used for treatment of CIRDs display antihyperglycemic properties by virtue of their anti-inflammatory, insulin-sensitizing, and/or insulinotropic effects. Figure 1, Figure 2 and Figure 3 illustrate the potential mechanisms underlying the antihyperglycemic properties exerted by csDMARDs, tsDMARDs, and bDMARDs, respectively. The evidence for substantial antidiabetic actions of DMARDs primarily comes from studies investigating the use of HCQ and IL-1 blocking therapies in diabetic and nondiabetic subjects with CIRDs. Of note, on the one hand, HCQ appears to exert antihyperglycemic properties mainly through insulin-sensitizing actions, with less evidence for insulinotropic effects. On the other hand, IL-1 blocking therapies (particularly anakinra) seem to act as antidiabetic drugs by improving beta cell dysfunction induced by a vicious cycle involving the release of excessive amounts of IL-1β from pancreatic beta cells in response to hyperglycemia and additional stimuli such as the generation of IAPP aggregates and increased circulating FFA levels. In this regard, IL-1 receptor blockade may also be proposed as a short-term pharmacological approach to restoring glucose homeostasis following metabolic relapses in diabetic patients with CIRDs.

TNF inhibitors appear to mainly act as insulin-sensitizing agents by attenuating TNF-α-induced insulin resistance, particularly in patients with a high degree of insulin resistance. This may result in reduced risk of developing diabetes in nondiabetic subjects and in improved glucose control in diabetic subjects, although larger prospective studies are needed to confirm such effects.

There is only little evidence for a possible antihyperglycemic role of IL-6 inhibitors, which may act as antihyperglycemic agents by reducing IL-6-driven insulin resistance. Other DMARDs such as abatacept may improve whole body insulin sensitivity by counteracting the recruitment or activation of adipose tissue-infiltrating immune cells. Finally, the antidiabetic actions of other DMARDs, such as leflunomide and JAKIs, have mainly been investigated in animal studies.

In light of these remarks, some DMARDs (e.g., HCQ and anakinra) could be alternatively termed “diabetes-modifying antirheumatic drugs”, since they may be repurposed for co-treatment of rheumatic diseases and comorbid diabetes. It is also worth specifying that different subtypes of T2D have recently been identified based on the main pathophysiological processes underlying the derangement of glucose metabolism. Among the subtypes of non-autoimmune diabetes mellitus, there are severe insulin-deficient diabetes, severe insulin-resistant diabetes, and combined insulin-resistant and insulin-deficient diabetes [209,210]. In this view, DMARDs may be classified based on their predominant mechanism of action on glucose homeostasis as follows: (i) insulin-sensitizing DMARDs such as TNF inhibitors, (ii) insulinotropic DMARDs such as IL-1 blocking therapies, and (iii) dual insulin-sensitizing and insulinotropic agents such as HCQ. This pharmacodynamics-driven classification may allow clinicians to use DMARDs in a tailored manner. This means that the rationale for the choice of the most appropriate DMARD in patients with CIRDs and comorbid diabetes relies not only on the specific rheumatic disease, but also on the main pathophysiological mechanism responsible for the derangement of glucose homeostasis.

Despite the aforementioned promising effects of some DMARDs on glucose homeostasis in patients with CIRDs (with or without diabetes), the observed changes in HbA1c values were rather modest in most studies. However, such studies were mainly observational or short-term intervention studies. In addition, it is worth reminding that HbA1c reflects the mean glycemia over the previous 8–12 weeks and remains to be the gold standard for assessing glucose control in patients with diabetes in the context of a normal hematological profile and in the nonpregnant population. Indeed, many conditions limit the accuracy of HbA1c as a reliable marker of average glucose, including untreated iron deficiency (with and without anemia), hemoglobin variants, altered life span or turnover of red blood cells, hemolysis, splenomegaly, red blood cell transfusions, and use of certain drugs (e.g., erythropoietin, dapsone, and iron) [211]. Moreover, HbA1c fails to address hypoglycemia and does not reflect daily glucose fluctuations and glucose variability over the previous 3 months [212]. These limitations can be overcome by subcutaneous continuous glucose monitoring (CGM) devices, which offer an advanced method to assess daily patterns of hyperglycemia and hypoglycemia, glucose excursions, as well as glucose variability [212]. Thus, CGM may represent a valid tool for assessing the true glucose-lowering effects of various DMARDs, particularly when these drugs can cause falsely low HbA1c values due to drug-induced hematological changes (e.g., SSZ) [103,104].

Interestingly, several DMARDs have also been studied as investigational therapies for the treatment of the coronavirus disease 2019 (COVID-19) caused by the severe acute respiratory syndrome coronavirus 2 (SARS-CoV-2) [213,214]. In the most severe cases of COVID-19, a dysregulated immune response called “cytokine storm” and characterized by an exuberant increase in circulating levels of proinflammatory cytokines (e.g., IL-1, IL-2, IL-6, TNF, and IFN-γ) leads to acute respiratory distress syndrome, disseminated intravascular coagulation, multiorgan failure, and ultimately death [215,216]. Some of these drugs (such as anakinra, tocilizumab, and baricitinib) showed protective effects against COVID-19-related hyperinflammatory state and mortality [217,218,219,220], whereas other agents (such as HCQ and CQ) have been proven ineffective and even associated with potential serious adverse events [221,222]. Since the beginning of the COVID-19 pandemic in March 2020, type 2 diabetes has emerged as an independent risk factor for adverse clinical outcomes and mortality in patients with COVID-19 [223,224,225,226]. Initially, there was also a concern among rheumatologists that patients with rheumatic diseases may be at increased risk for COVID-19-related morbidity and mortality; however, data have shown that the main drivers for worse clinical outcomes in patients with rheumatic diseases are male sex, age, and comorbidities such as diabetes and CVD [227,228]. With this regard, some DMARDs may provide additional clinical benefits to patients with CIRDs and comorbid diabetes affected by COVID-19, resulting in a substantial reduction in morbidity and mortality related to SARS-CoV-2 infection.

## 7. Conclusions

The evidence suggests that targeting inflammation may be a valid therapeutic approach in the management of CIRDs associated with comorbid T2D. In this view, DMARDs are promising drug candidates that may potentially reduce rheumatic disease activity and severity, ameliorate glucose control, and prevent the development of diabetes-associated cardiovascular complications and metabolic dysfunctions at the same time. Mechanistic studies are needed to better understand the exact mechanism of action underlying the antihyperglycemic properties of different DMARDs. Moreover, there is need for future randomized controlled trials conducted on patients with CIRDs and comorbid T2D to confirm the beneficial metabolic and cardiovascular effects as well as the safety profile of distinct DMARDs in the long term. Prospective studies are also warranted to clarify whether these drugs are able to reduce the risk for developing prediabetes and diabetes in nondiabetic patients with CIRDs.

## Data Availability

Not applicable.

## References

[1] Fowler J.M. (2008). Microvascular and Macrovascular Complications of Diabetes. Clin. Diabetes Apr..

[2] Wright A.K., Kontopantelis E., Emsley R., Buchan I., Mamas M.A., Sattar N., Ashcroft D., Rutter M.K. (2019). Cardiovascular Risk and Risk Factor Management in Type 2 Diabetes Mellitus. Circulation.

[3] Saeedi P., Petersohn I., Salpea P., Malanda B., Karuranga S., Unwin N., Colagiuri S., Guariguata L., Motala A.A., Ogurtsova K. (2019). Global and regional diabetes prevalence estimates for 2019 and projections for 2030 and 2045: Results from the International Diabetes Federation Diabetes Atlas. Diabetes Res. Clin. Pract..

[4] American Diabetes Association (2021). Classification and Diagnosis of Diabetes: Standards of Medical Care in Diabetes—2020. Diabetes Care.

[5] Adeva-Andany M.M., Martínez-Rodríguez J., González-Lucán M., Fernández-Fernández C., Castro-Quintela E. (2019). Insulin resistance is a cardiovascular risk factor in humans. Diabetes Metab. Syndr..

[6] Nguyen D.V., Shaw L.C., Grant M.B. (2012). Inflammation in the pathogenesis of microvascular complications in diabetes. Front Endocrinol..

[7] Donath M.Y., Dinarello C.A., Mandrup-Poulsen T. (2019). Targeting innate immune mediators in type 1 and type 2 diabetes. Nat. Rev. Immunol..

[8] Nunemaker C.S. (2016). Considerations for Defining Cytokine Dose, Duration, and Milieu That Are Appropriate for Modeling Chronic Low-Grade Inflammation in Type 2 Diabetes. J. Diabetes Res..

[9] Pickup J.C., Mattock M.B., Chusney G.D., Burt D. (1997). NIDDM as a disease of the innate immune system: Association of Acute-Phase Reactants and Interleukin-6 with Metabolic Syndrome, X. Diabetologia.

[10] Spranger J., Kroke A., Möhlig M., Hoffmann K., Bergmann M.M., Ristow M., Boeing H., Pfeiffer A.F.H. (2003). Inflammatory cytokines and the risk to develop type 2 diabetes: Results of the Prospective Population-Based European Prospective Investigation into Cancer and Nutrition (EPIC)-Potsdam Study. Diabetes.

[11] Herder C., Illig T., Rathmann W., Martin S., Haastert B., Müller-Scholze S., Holle R., Thorand B., Koenig W., Wichmann H.E. (2005). Inflammation and type 2 diabetes: Results from KORA Augsburg. Gesundheitswesen.

[12] Herder C., Brunner E.J., Rathmann W., Strassburger K., Tabák A.G., Schloot N.C., Witte D.R. (2009). Elevated levels of the anti-inflammatory interleukin-1 receptor antagonist precede the onset of type 2 diabetes: The Whitehall II Study. Diabetes Care.

[13] Pradhan A.D., Manson J.E., Rifai N., Buring J.E., Ridker P.M. (2001). C-reactive protein, interleukin 6, and risk of developing type 2 diabetes mellitus. JAMA.

[14] Liang W., Ye D.D. (2019). The potential of adipokines as biomarkers and therapeutic agents for vascular complications in type 2 diabetes mellitus. Cytokine Growth Factor Rev..

[15] Calle M.C., Fernandez M.L. (2012). Inflammation and type 2 diabetes. Diabetes Metab..

[16] Donath M.Y., Shoelson S.E. (2011). Type 2 diabetes as an inflammatory disease. Nat. Rev. Immunol..

[17] Böni-Schnetzler M., Meier D.T. (2019). Islet inflammation in type 2 diabetes. Semin Immunopathol..

[18] Eguchi K., Nagai R. (2017). Islet inflammation in type 2 diabetes and physiology. J. Clin. Investig..

[19] Park Y.M., Myers M., Vieira-Potter V.J. (2014). Adipose tissue inflammation and metabolic dysfunction: Role of Exercise. Mo. Med..

[20] Tam L.S., Tomlinson B., Chu T.T., Li M., Leung Y.Y., Kwok L.W., Li T.K., Yu T., Zhu Y.-E., Wong K.-C. (2008). Cardiovascular risk profile of patients with psoriatic arthritis compared to controls—The role of inflammation. Rheumatology.

[21] Seriolo B., Ferrone C., Cutolo M. (2008). Longterm anti-tumor necrosis factor-alpha treatment in patients with refractory rheumatoid arthritis: Relationship between Insulin Resistance and Disease Activity. J. Rheumatol..

[22] Ruscitti P., Ursini F., Cipriani P., Ciccia F., Liakouli V., Carubbi F., Guggino G., Berardicurti O., Grembiale R., Triolo G. (2017). Prevalence of type 2 diabetes and impaired fasting glucose in patients affected by rheumatoid arthritis: Results from a cross-sectional study. Medicine.

[23] Sidiropoulos P.I., Karvounaris S.A., Boumpas D.T. (2008). Metabolic syndrome in rheumatic diseases: Epidemiology, Pathophysiology, and Clinical Implications. Arthritis Res. Ther..

[24] Ziade N., El Khoury B., Zoghbi M., Merheb G., Karam G.A., Mroue’ K., Messaykeh J. (2020). Prevalence and pattern of comorbidities in chronic rheumatic and musculoskeletal diseases: The Comord Study. Sci. Rep..

[25] Jiang P., Li H., Li X. (2015). Diabetes mellitus risk factors in rheumatoid arthritis: A Systematic Review and Meta-Analysis. Clin. Exp. Rheumatol..

[26] Albrecht K., Luque Ramos A., Hoffmann F., Redeker I., Zink A. (2018). High prevalence of diabetes in patients with rheumatoid arthritis: Results from a Questionnaire Survey Linked to Claims Data. Rheumatology.

[27] Dal Bello G., Gisondi P., Idolazzi L., Girolomoni G. (2020). Psoriatic Arthritis and Diabetes Mellitus: A Narrative Review. Rheumatol. Ther..

[28] Ruscitti P., Ursini F., Cipriani P., Liakouli V., Carubbi F., Berardicurti O., De Sarro G., Giacomelli R. (2017). Poor clinical response in rheumatoid arthritis is the main risk factor for diabetes development in the short-term: A 1-Year, Single-Centre, Longitudinal Study. PLoS ONE.

[29] Baker J.F., England B.R., George M., Cannon G., Sauer B., Ogdie A., Hamilton B.C., Hunter C., Duryee M.J., Thiele G. (2021). Disease activity, cytokines, chemokines and the risk of incident diabetes in rheumatoid arthritis. Ann. Rheum. Dis..

[30] Ozen G., Pedro S., Holmqvist M.E., Avery M., Wolfe F., Michaud K. (2017). Risk of diabetes mellitus associated with disease-modifying antirheumatic drugs and statins in rheumatoid arthritis. Ann. Rheum. Dis..

[31] Solomon D.H., Karlson E.W., Rimm E.B., Cannuscio C.C., Mandl L.A., Manson J.E., Stampfer M.J., Curhan G.C. (2003). Cardiovascular morbidity and mortality in women diagnosed with rheumatoid arthritis. Circulation.

[32] del Rincón I.D., Williams K., Stern M.P., Freeman G.L., Escalante A. (2001). High incidence of cardiovascular events in a rheumatoid arthritis cohort not explained by traditional cardiac risk factors. Arthritis Rheum..

[33] Young A., Koduri G., Batley M., Kulinskaya E., Gough A., Norton S., Dixey J. (2007). Mortality in rheumatoid arthritis. Increased in the early course of disease, in ischaemic heart disease and in pulmonary fibrosis. Rheumatology.

[34] Goodson N., Marks J., Lunt M., Symmons D. (2005). Cardiovascular admissions and mortality in an inception cohort of patients with rheumatoid arthritis with onset in the 1980s and 1990s. Ann. Rheum. Dis..

[35] Zeller C.B., Appenzeller S. (2008). Cardiovascular disease in systemic lupus erythematosus: The Role of Traditional and Lupus Related Risk Factors. Curr. Cardiol. Rev..

[36] Bartoloni E., Baldini C., Schillaci G., Quartuccio L., Priori R., Carubbi F., Bini V., Alunno A., Bombardieri S., De Vita S. (2015). Cardiovascular disease risk burden in primary Sjögren’s syndrome: Results of a Population-Based Multicentre Cohort Study. J. Intern. Med..

[37] Aviña-Zubieta J.A., Choi H.K., Sadatsafavi M., Etminan M., Esdaile J.M., Lacaille D. (2008). Risk of cardiovascular mortality in patients with rheumatoid arthritis: A Meta-Analysis of Observational Studies. Arthritis Rheum..

[38] Meune C., Touzé E., Trinquart L., Allanore Y. (2009). Trends in cardiovascular mortality in patients with rheumatoid arthritis over 50 years: A Systematic Review and Meta-Analysis of Cohort Studies. Rheumatology.

[39] Lau C.S., Chia F., Harrison A., Hsieh T.Y., Jain R., Jung S.M., Kishimoto M., Kumar A., Leong K.P., Li Z. (2015). APLAR rheumatoid arthritis treatment recommendations. Int. J. Rheum. Dis..

[40] Bartels C.M., Buhr K.A., Goldberg J.W., Bell C.L., Visekruna M., Nekkanti S., Greenlee R.T. (2014). Mortality and cardiovascular burden of systemic lupus erythematosus in a US population-based cohort. J. Rheumatol..

[41] Goodson N.J., Wiles N.J., Lunt M., Barrett E.M., Silman A.J., Symmons D.P. (2002). Mortality in early inflammatory polyarthritis: Cardiovascular Mortality is Increased in Seropositive Patients. Arthritis Rheum..

[42] Navarro-Cano G., Del Rincón I., Pogosian S., Roldán J.F., Escalante A. (2003). Association of mortality with disease severity in rheumatoid arthritis, independent of comorbidity. Arthritis Rheum..

[43] Maradit-Kremers H., Crowson C.S., Nicola P.J., Ballman K.V., Roger V.L., Jacobsen S.J., Gabriel S.E. (2005). Increased unrecognized coronary heart disease and sudden deaths in rheumatoid arthritis: A Population-Based Cohort Study. Arthritis Rheum..

[44] Motta F., Sica A., Selmi C. (2020). Frailty in Rheumatic Diseases. Front. Immunol..

[45] Del Rincón I., Williams K., Stern M.P., Freeman G.L., O’Leary D.H., Escalante A. (2003). Association between carotid atherosclerosis and markers of inflammation in rheumatoid arthritis patients and healthy subjects. Arthritis Rheum..

[46] Mäki-Petäjä K.M., Hall F.C., Booth A.D., Wallace S.M., Yasmin, Bearcroft P.W., Harish S., Furlong A., McEniery C.M., Brown J. (2006). Rheumatoid arthritis is associated with increased aortic pulse-wave velocity, which is reduced by anti-tumor necrosis factor-alpha therapy. Circulation.

[47] Kerekes G., Szekanecz Z., Dér H., Sándor Z., Lakos G., Muszbek L., Csipö I., Sipka S., Seres I., Paragh G. (2008). Endothelial dysfunction and atherosclerosis in rheumatoid arthritis: A Multiparametric Analysis Using Imaging Techniques and Laboratory Markers of Inflammation and Autoimmunity. J. Rheumatol..

[48] Rho Y.H., Oeser A., Chung C.P., Milne G.L., Stein C.M. (2009). Drugs Used in the Treatment of Rheumatoid Arthritis: Relationship between Current Use and Cardiovascular Risk Factors. Arch. Drug Inf..

[49] Verma A.K., Bhatt D., Goyal Y., Dev K., Beg M.M.A., Alsahli M.A., Rahmani A.H. (2021). Association of Rheumatoid Arthritis with Diabetic Comorbidity: Correlating Accelerated Insulin Resistance to Inflammatory Responses in Patients. J. Multidiscip. Healthc..

[50] Velikova T.V., Kabakchieva P.P., Assyov Y.S., Georgiev T. (2021). Targeting Inflammatory Cytokines to Improve Type 2 Diabetes. Control Biomed. Res. Int..

[51] Benjamin O., Bansal P., Goyal A., Lappin S.L. Disease Modifying Anti-Rheumatic Drugs (DMARD); StatPearls: Treasure Island, FL, USA. https://www.ncbi.nlm.nih.gov/books/NBK507863/.

[52] Smolen J.S., van der Heijde D., Machold K.P., Aletaha D., Landewé R. (2014). Proposal for a new nomenclature of disease-modifying antirheumatic drugs. Ann. Rheum. Dis..

[53] Cronstein B.N., Aune T.M. (2020). Methotrexate and its mechanisms of action in inflammatory arthritis. Nat. Rev. Rheumatol..

[54] Banerjee D., Ercikan-Abali E., Waltham M., Schnieders B., Hochhauser D., Li W.W., Fan J., Gorlick R., Goker E., Bertino J.R. (1995). Molecular mechanisms of resistance to antifolates, a review. Acta Biochim. Pol..

[55] Perdan-Pirkmajer K., Pirkmajer S., Thevis M., Thomas A., Praprotnik S., Hočevar A., Rotar Ž., Gašperšič N., Sodin-Šemrl S., Žibert J. (2016). Methotrexate reduces HbA1c concentration but does not produce chronic accumulation of ZMP in patients with rheumatoid or psoriatic arthritis. Scand. J. Rheumatol..

[56] Baghdadi L.R. (2020). Effect of methotrexate use on the development of type 2 diabetes in rheumatoid arthritis patients: A Systematic Review and Meta-Analysis. PLoS ONE.

[57] Mantravadi S., George M., Brensinger C., Du M., Baker J.F., Ogdie A. (2020). Impact of tumor necrosis factor inhibitors and methotrexate on diabetes mellitus among patients with inflammatory arthritis. BMC Rheumatol..

[58] Sotoudehmanesh R., Anvari B., Akhlaghi M., Shahraeeni S., Kolahdoozan S. (2010). Methotrexate hepatotoxicity in patients with rheumatoid arthritis. Middle East J. Dig. Dis..

[59] Baghdadi L.R., Woodman R.J., Shanahan E.M., Wiese M.D., Mangoni A.A. (2018). Genetic polymorphism of the methotrexate transporter ABCG2, blood pressure and markers of arterial function in patients with rheumatoid arthritis: Repeated Cross-Sectional Study. Pharmgenom. Pers. Med..

[60] Chan E.S., Cronstein B.N. (2013). Mechanisms of action of methotrexate. Bull. Hosp. Jt. Dis..

[61] Hardie D.G. (2013). AMPK: A Target for Drugs and Natural Products with Effects on Both Diabetes and Cancer. Diabetes.

[62] Corton J.M., Gillespie J.G., Hawley S.A., Hardie D.G. (1995). 5-aminoimidazole-4-carboxamide ribonucleoside. A specific method for activating AMP-activated protein kinase in intact cells?. Eur. J. Biochem..

[63] Viollet B., Foretz M., Guigas B., Horman S., Dentin R., Bertrand L., Hue L., Andreelli F. (2006). Activation of AMP-activated protein kinase in the liver: A New Strategy for the Management of Metabolic Hepatic Disorders. J. Physiol..

[64] Musi N., Goodyear L.J. (2003). AMP-activated protein kinase and muscle glucose uptake. Acta Physiol. Scand..

[65] Zhou G., Myers R., Li Y., Chen Y., Shen X., Fenyk-Melody J., Wu M., Ventre J., Doebber T., Fujii N. (2001). Role of AMP-activated protein kinase in mechanism of metformin action. J. Clin. Investig..

[66] Rena G., Hardie D.G., Pearson E.R. (2017). The mechanisms of action of metformin. Diabetologia.

[67] Pirkmajer S., Kulkarni S.S., Tom R.Z., Ross F.A., Hawley S.A., Hardie D.G., Zierath J.R., Chibalin A.V. (2015). Methotrexate promotes glucose uptake and lipid oxidation in skeletal muscle via AMPK activation. Diabetes.

[68] Schrezenmeier E., Dörner T. (2020). Mechanisms of action of hydroxychloroquine and chloroquine: Implications for Rheumatology. Nat. Rev. Rheumatol..

[69] Lotteau V., Teyton L., Peleraux A., Nilsson T., Karlsson L., Schmid S., Quaranta V., Peterson A.P. (1990). Intracellular transport of class II MHC molecules directed by invariant chain. Nature.

[70] Kuznik A., Bencina M., Svajger U., Jeras M., Rozman B., Jerala R. (2011). Mechanism of endosomal TLR inhibition by antimalarial drugs and imidazoquinolines. J. Immunol..

[71] Savarino A., Boelaert J.R., Cassone A., Majori G., Cauda R. (2003). Effects of chloroquine on viral infections: An Old Drug Against Today’s Diseases?. Lancet Infect. Dis..

[72] van den Borne B.E., Dijkmans B.A., de Rooij H.H., le Cessie S., Verweij C.L. (1997). Chloroquine and hydroxychloroquine equally affect tumor necrosis factor-alpha, interleukin 6, and interferon-gamma production by peripheral blood mononuclear cells. J. Rheumatol..

[73] Wondafrash D.Z., Desalegn T.Z., Yimer E.M., Tsige A.G., Adamu B.A., Zewdie K.A. (2020). Potential Effect of Hydroxychloroquine in Diabetes Mellitus: A Systematic Review on Preclinical and Clinical Trial Studies. J. Diabetes Res..

[74] Infante M., Ricordi C., Fabbri A. (2020). Antihyperglycemic properties of hydroxychloroquine in patients with diabetes: Risks and Benefits at the Time of COVID-19 Pandemic. J. Diabetes.

[75] Wasko M.C.M., Hubert H.B., Lingala V.B., Elliott J.R., Luggen M.E., Fries J.F., Ward M.M. (2007). Hydroxychloroquine and risk of diabetes in patients with rheumatoid arthritis. JAMA.

[76] Bili A., Sartorius J.A., Kirchner H.L., Morris S.J., Ledwich L.J., Antohe J.L., Dancea S., Newman E.D., Wasko M.C.M. (2011). Hydroxychloroquine use and decreased risk of diabetes in rheumatoid arthritis patients. J. Clin. Rheumatol..

[77] Solomon D.H., Massarotti E., Garg R., Liu J., Canning C., Schneeweiss S. (2011). Association between disease-modifying antirheumatic drugs and diabetes risk in patients with rheumatoid arthritis and psoriasis. JAMA.

[78] Chen H.H., Chen D.Y., Lin C.C., Chen Y.M., Lai K.L., Lin C.H. (2017). Association between use of disease-modifying antirheumatic drugs and diabetes in patients with ankylosing spondylitis, rheumatoid arthritis, or psoriasis/psoriatic arthritis: A Nationwide, Population-Based Cohort Study of 84,989 Patients. Ther. Clin. Risk Manag..

[79] Chen Y.-M., Lin C.-H., Lan T.-H., Chen H.-H., Chang S.-N., Wang J.-S., Hung W.-T., Lan J.-L., Chen D.-Y. (2015). Hydroxychloroquine reduces risk of incident diabetes mellitus in lupus patients in a dose-dependent manner: A Population-Based Cohort Study. Rheumatology.

[80] Chen T.H., Lai T.Y., Wang Y.H., Chiou J.Y., Hung Y.M., Wei J.C. (2019). Hydroxychloroquine was associated with reduced risk of new-onset diabetes mellitus in patients with Sjögren syndrome. QJM.

[81] Quatraro A., Consoli G., Magno M., Caretta F., Nardozza A., Ceriello A., Giugliano D. (1990). Hydroxychloroquine in decompensated, treatment-refractory noninsulin-dependent diabetes mellitus. A new job for an old drug?. Ann. Intern. Med..

[82] Gerstein H.C., Thorpe K.E., Taylor D.W., Haynes R.B. (2002). The effectiveness of hydroxychloroquine in patients with type 2 diabetes mellitus who are refractory to sulfonylureas—A randomized trial. Diabetes Res. Clin. Pract..

[83] Hsia S.H., Duran P., Lee M.L., Davidson M.B. (2020). Randomized controlled trial comparing hydroxychloroquine with pioglitazone as third-line agents in type 2 diabetic patients failing metformin plus a sulfonylurea: A Pilot Study. Diabetes.

[84] Rekedal L.R., Massarotti E., Garg R., Bhatia R., Gleeson T., Lu B., Solomon D.H. (2010). Changes in glycosylated hemoglobin after initiation of hydroxychloroquine or methotrexate treatment in diabetes patients with rheumatic diseases. Arthritis Care Res..

[85] Simental-Mendía L.E., Simental-Mendía M., Sánchez-García A., Linden-Torres E. (2021). Effect of hydroxychloroquine on glucose control in patients with and without diabetes: A Systematic Review and Meta-Analysis of Randomized Controlled Clinical Trials. Eur. J. Clin. Pharmacol..

[86] Gupta A. (2019). Real-World Clinical Effectiveness and Tolerability of Hydroxychloroquine 400 Mg in Uncontrolled Type 2 Diabetes Subjects who are not Willing to Initiate Insulin Therapy (HYQ-Real-World Study). Curr. Diabetes Rev..

[87] Pareek A., Chandurkar N., Thomas N., Viswanathan V., Deshpande A., Gupta O., Shah A., Kakrani A., Bhandari S., Thulasidharan N. (2014). Efficacy and safety of hydroxychloroquine in the treatment of type 2 diabetes mellitus: A Double Blind, Randomized Comparison with Pioglitazone. Curr. Med. Res. Opin..

[88] Purkait I., Pareek A., Panneerselvam A., Mukhopadhyay M.K., Kumar S., Chandratreya S.A. (2019). 1189-P: Effectiveness of Hydroxychloroquine (HCQ) 400 mg in Uncontrolled T2D Patients on Dual Therapy of Metformin and Sulfonylurea: A Real-World Experience in India. Diabetes.

[89] Das A.K., Kalra S., Tiwaskar M., Bajaj S., Seshadri K., Chowdhury S., Sahay R., Indurkar S., Unnikrishnan A.G., Phadke U. (2019). Expert Group Consensus Opinion: Role of Anti-inflammatory Agents in the Management of Type-2 Diabetes (T2D). J. Assoc. Physicians India.

[90] Chakravarti H.N., Nag A. (2021). Efficacy and safety of hydroxychloroquine as add-on therapy in uncontrolled type 2 diabetes patients who were using two oral antidiabetic drugs. J. Endocrinol. Investig..

[91] Wasko M.C.M., McClure C.K., Kelsey S.F., Huber K., Orchard T., Toledo F.G.S. (2015). Antidiabetogenic effects of hydroxychloroquine on insulin sensitivity and beta cell function: A randomised trial. Diabetologia.

[92] Powrie J.K., Smith G.D., Shojaee-Moradie F., Sonksen P.H., Jones R.H. (1991). Mode of action of chloroquine in patients with non-insulin-dependent diabetes mellitus. Am. J. Physiol. Metab..

[93] Toledo F.G.S., Miller R.G., Helbling N.L., Zhang Y., DeLany J.P. (2021). The effects of hydroxychloroquine on insulin sensitivity, insulin clearance and inflammation in insulin-resistant adults: A Randomized Trial. Diabetes Obes. Metab..

[94] Jorge A., Lu N., Choi H., Esdaile J.M., Lacaille D., Avina-Zubieta J.A. (2021). Hydroxychloroquine Use and Cardiovascular Events Among Patients with Systemic Lupus Erythematosus and Rheumatoid Arthritis. Arthritis Care Res..

[95] Liu D., Li X., Zhang Y., Kwong J.S.-W., Li L., Zhang Y., Xu C., Li Q., Sun X., Tian H. (2018). Chloroquine and hydroxychloroquine are associated with reduced cardiovascular risk: A systematic review and meta-analysis. Drug Des. Dev. Ther..

[96] Floris A., Piga M., Mangoni A.A., Bortoluzzi A., Erre G.L., Cauli A. (2018). Protective Effects of Hydroxychloroquine against Accelerated Atherosclerosis in Systemic Lupus Erythematosus. Mediat. Inflamm..

[97] Schmidt-Tanguy A., Voswinkel J., Henrion D., Subra J.F., Loufrani L., Rohmer V., Ifrah N., Belizna C. (2013). Antithrombotic effects of hydroxychloroquine in primary antiphospholipid syndrome patients. J. Thromb. Haemost..

[98] Qiao X., Zhou Z.-C., Niu R., Su Y.-T., Sun Y., Liu H.-L., Teng J.-L., Ye J.-N., Shi H., Yang C.-D. (2019). Hydroxychloroquine Improves Obesity-Associated Insulin Resistance and Hepatic Steatosis by Regulating Lipid Metabolism. Front. Pharmacol..

[99] La Montagna G., Cacciapuoti F., Buono R., Manzella D., Mennillo G.A., Arciello A., Valentini G., Paolisso G. (2007). Insulin resistance is an independent risk factor for atherosclerosis in rheumatoid arthritis. Diabetes Vasc. Dis. Res..

[100] Choi J., Fenando A. Sulfasalazine; StatPearls: Treasure Island, FL, USA. https://www.ncbi.nlm.nih.gov/books/NBK557809/..

[101] Wahl C., Liptay S., Adler G., Schmid R.M. (1998). Sulfasalazine: A Potent and Specific Inhibitor of Nuclear Factor Kappa B. J. Clin. Investig..

[102] Haas R.M., Li P., Chu J.W. (2005). Glucose-Lowering Effects of Sulfasalazine in Type 2 Diabetes. Diabetes Care.

[103] Mitchell K., Mukhopadhyay B. (2018). Drug-Induced Falsely Low A1C: Report of a Case Series From a Diabetes Clinic. Clin. Diabetes.

[104] Tack C.J., Wetzels J.F. (1996). Decreased HbA1c Levels Due to Sulfonamide-Induced Hemolysis in Two IDDM Patients. Diabetes Care.

[105] Kesson C.M., Whitelaw J.W., Ireland J.T. (1979). Drug-induced haemolysis and fast haemoglobin A1 in diabetes mellitus. BMJ.

[106] Lai Y.-C., Wang C.-S., Wang Y.-C., Hsu Y.-L., Chuang L.-M. (2012). Falsely decreased HbA1c in a type 2 diabetic patient treated with dapsone. J. Formos. Med. Assoc..

[107] N’Dow S.M.S., Donnelly L.A., Pearson E.R., Rena G. (2020). In a cohort of individuals with type 2 diabetes using the drug sulfasalazine, HbA 1c lowering is associated with haematological changes. Diabet. Med..

[108] Padda I.S., Goyal A. Leflunomide; StatPearls: Treasure Island, FL, USA. https://www.ncbi.nlm.nih.gov/books/NBK557799/.

[109] Fox R.I., Herrmann M.L., Frangou C.G., Wahl G.M., Morris R.E., Strande V., Kirschbaum B.J. (1999). Mechanism of Action for Leflunomide in Rheumatoid Arthritis. Clin. Immunol..

[110] Doscas M.E., Williamson A.J., Usha L., Bogachkov Y., Rao G.S., Xiao F., Wang Y., Ruby C., Kaufman H., Zhou J. (2014). Inhibition of p70 S6 Kinase (S6K1) Activity by A77 1726 and Its Effect on Cell Proliferation and Cell Cycle Progress. Neoplasia.

[111] Chen J., Sun J., Doscas M.E., Ye J., Williamson A.J., Li Y., Li Y., Prinz R.A., Xu X. (2018). Control of hyperglycemia in male mice by leflunomide: Mechanisms of Action. J. Endocrinol..

[112] Zerilli T., Ocheretyaner E. (2015). Apremilast (Otezla): A New Oral Treatment for Adults With Psoriasis and Psoriatic Arthritis. Pharm. Ther..

[113] Schafer P. (2012). Apremilast mechanism of action and application to psoriasis and psoriatic arthritis. Biochem. Pharmacol..

[114] Kumar N., Goldminz A.M., Kim N., Gottlieb A.B. (2013). Phosphodiesterase 4-targeted treatments for autoimmune diseases. BMC Med..

[115] Houslay M.D., Schafer P., Zhang K.Y. (2005). Keynote review: Phosphodiesterase-4 as a therapeutic target. Drug Discov. Today.

[116] Mazzilli S., Lanna C., Chiaramonte C., Cesaroni G.M., Zangrilli A., Palumbo V., Cosio T., Dattola A., Gaziano R., Galluzzo M. (2020). Real life experience of apremilast in psoriasis and arthritis psoriatic patients: Preliminary Results on Metabolic Biomarkers. J. Dermatol..

[117] Puig L., Korman N., Greggio C., Cirulli J., Chandran V., Khraishi M., Meht N.N. (2018). Hemoglobin A1c and weight changes with apremilast in patients with psoriasis and psoriatic arthritis: Pooled Laboratory Analysis of the Phase 3 Esteem and Palace Trials. J. Am. Acad. Derm..

[118] Lanna C., Cesaroni G.M., Mazzilli S., Bianchi L., Campione E. (2019). Small Molecules, Big Promises: Improvement of Psoriasis Severity and Glucidic Markers with Apremilast: A Case Report. Diabetes Metab. Syndr. Obesity Targets Ther..

[119] Puig L., Korman N., Greggio C., Cirulli J., Teng L., Chandran V., Khraishi M., Paris M., Mehta N.N. (2019). Long-term hemoglobin A1c changes with apremilast in patients with psoriasis and psoriatic arthritis: Pooled Analysis of Phase 3 Esteem and Palace Trials and Phase 3b Liberate Trial. J. Am. Acad. Dermatol..

[120] Wouters E.F.M., Bredenbroker D., Teichmann P., Brose M., Rabe K.F., Fabbri L.M., Göke B. (2012). Effect of the Phosphodiesterase 4 Inhibitor Roflumilast on Glucose Metabolism in Patients with Treatment-Naive, Newly Diagnosed Type 2 Diabetes Mellitus. J. Clin. Endocrinol. Metab..

[121] Heimann E., Jones H.A., Resjö S., Manganiello V.C., Stenson L., Degerman E. (2010). Expression and Regulation of Cyclic Nucleotide Phosphodiesterases in Human and Rat Pancreatic Islets. PLoS ONE.

[122] Pyne N., Furman B.L. (2003). Cyclic nucleotide phosphodiesterases in pancreatic islets. Diabetologia.

[123] Muo I.M., MacDonald S.D., Madan R., Park S.-J., Gharib A.M., Martinez P.E., Walter M.F., Yang S.B., Rodante J.A., Courville A.B. (2019). Early effects of roflumilast on insulin sensitivity in adults with prediabetes and overweight/obesity involve age-associated fat mass loss—Results of an exploratory study. Diabetes Metab. Syndr. Obes. Targets Ther..

[124] Armani A., Marzolla V., Rosano G.M., Fabbri A., Caprio M. (2011). Phosphodiesterase type 5 (PDE5) in the adipocyte: A Novel Player in Fat Metabolism?. Trends Endocrinol. Metab..

[125] Cada D.J., Demaris K., Levien T.L., Baker D.E. (2013). Tofacitinib. Hosp. Pharm..

[126] Al-Salama Z.T., Scott L.J. (2018). Baricitinib: A Review in Rheumatoid Arthritis. Drugs.

[127] Dodington D.W., Desai H.R., Woo M. (2018). JAK/STAT—Emerging Players in Metabolism. Trends Endocrinol. Metab..

[128] Bako H.Y., Ibrahim M.A., Isah M.S., Ibrahim S. (2019). Inhibition of JAK-STAT and NF-κB signalling systems could be a novel therapeutic target against insulin resistance and type 2 diabetes. Life Sci..

[129] Collotta D., Hull W., Mastrocola R., Chiazza F., Cento A.S., Murphy C., Verta R., Alves G.F., Gaudioso G., Fava F. (2020). Baricitinib counteracts metaflammation, thus protecting against diet-induced metabolic abnormalities in mice. Mol. Metab..

[130] Marrero M.B., Banes-Berceli A.K., Stern D.M., Eaton D. (2006). Role of the JAK/STAT signaling pathway in diabetic nephropathy. Am. J. Physiol. Physiol..

[131] Tuttle K.R., Brosius F.C., Adler S.G., Kretzler M., Mehta R.L., Tumlin J.A., Tanaka Y., Handea M., Liu J., Slik M.E. (2018). JAK1/JAK2 inhibition by baricitinib in diabetic kidney disease: Results from a Phase 2 Randomized Controlled Clinical Trial. Nephrol. Dial. Transplant..

[132] IIdriss H.T., Naismith J.H. (2000). TNF alpha and the TNF receptor superfamily: Structure-Function Relationship(s). Microsc. Res. Tech..

[133] Hotamisligil G.S., Peraldi P., Budavari A., Ellis R., White M.F., Spiegelman B.M. (1996). IRS-1-Mediated Inhibition of Insulin Receptor Tyrosine Kinase Activity in TNF-α- and Obesity-Induced Insulin Resistance. Science.

[134] Akash M.S.H., Rehman K., Liaqat A. (2018). Tumor Necrosis Factor-Alpha: Role in Development of Insulin Resistance and Pathogenesis of Type 2 Diabetes Mellitus. J. Cell. Biochem..

[135] Coates L.C., FitzGerald O., Helliwell P.S., Paul C. (2016). Psoriasis, psoriatic arthritis, and rheumatoid arthritis: Is All Inflammation the Same?. Semin. Arthritis Rheum..

[136] Schett G., Coates L.C., Ash Z.R., Finzel S., Conaghan P.G. (2011). Structural damage in rheumatoid arthritis, psoriatic arthritis, and ankylosing spondylitis: Traditional Views, Novel Insights Gained from TNF Blockade, and Concepts for the Future. Arthritis Res. Ther..

[137] Schett G., Gravallese E. (2012). Bone erosion in rheumatoid arthritis: Mechanisms, Diagnosis and Treatment. Nat. Rev. Rheumatol..

[138] Gerriets V., Bansal P., Goyal A. Tumor Necrosis Factor Inhibitors; StatPearls: Treasure Island, FL, USA. https://www.ncbi.nlm.nih.gov/books/NBK482425/.

[139] Ma X., Xu S. (2013). TNF inhibitor therapy for rheumatoid arthritis. Biomed. Rep..

[140] Aguirre V., Werner E.D., Giraud J., Lee Y.H., Shoelson S.E., White M.F. (2002). Phosphorylation of Ser307 in insulin receptor substrate-1 blocks interactions with the insulin receptor and inhibits insulin action. J. Biol. Chem..

[141] Kanety H., Feinstein R., Papa M.Z., Hemi R., Karasik A. (1995). Tumor Necrosis Factor α-induced Phosphorylation of Insulin Receptor Substrate-1 (IRS-1). J. Biol. Chem..

[142] Uysal K.T., Wiesbrock S.M., Marino M.W., Hotamisligil G.S. (1997). Protection from obesity-induced insulin resistance in mice lacking TNF-α function. Nature.

[143] Stagakis I., Bertsias G., Karvounaris S., Kavousanaki M., Virla D., Raptopoulou A., Kardassis D., Boumpas D.T., Sidiropoulos P.I. (2012). Anti-tumor necrosis factor therapy improves insulin resistance, beta cell function and insulin signaling in active rheumatoid arthritis patients with high insulin resistance. Arthritis Res. Ther..

[144] Stavropoulos-Kalinoglou A., Metsios G.S., Panoulas V.F., Nightingale P., Koutedakis Y., Kitas G.D. (2012). Anti-tumour necrosis factor alpha therapy improves insulin sensitivity in normal-weight but not in obese patients with rheumatoid arthritis. Arthritis Res. Ther..

[145] Oguz F.M., Oguz A., Uzunlulu M. (2007). The effect of infliximab treatment on insulin resistance in patients with rheumatoid arthritis. Acta Clin. Belg..

[146] Méndez-García L.A., Trejo-Millán F., Martínez-Reyes C.P., Manjarrez-Reyna A.N., Esquivel-Velázquez M., Melendez-Mier G., Islas-Andrade S., Rojas-Bernabé A., Kzhyshkowska J., Escobedo G. (2018). Infliximab ameliorates tumor necrosis factor-alpha-induced insulin resistance by attenuating PTP1B activation in 3T3L1 adipocytes in vitro. Scand. J. Immunol..

[147] Kiortsis D.N., Mavridis A.K., Vasakos S., Nikas S.N., Drosos A.A. (2005). Effects of infliximab treatment on insulin resistance in patients with rheumatoid arthritis and ankylosing spondylitis. Ann. Rheum. Dis..

[148] Gupta-Ganguli M., Cox K., Means B., Gerling I., Solomon S.S. (2011). Does therapy with anti-TNF-alpha improve glucose tolerance and control in patients with type 2 diabetes?. Diabetes Care.

[149] Stanley T., Zanni M., Johnsen S., Rasheed S., Makimura H., Lee H., Khor V.K., Ahima R.S., Grinspoon S.K. (2011). TNF-α Antagonism with Etanercept Decreases Glucose and Increases the Proportion of High Molecular Weight Adiponectin in Obese Subjects with Features of the Metabolic Syndrome. J. Clin. Endocrinol. Metab..

[150] Bernstein L.E., Berry J., Kim S., Canavan B., Grinspoon S.K. (2006). Effects of Etanercept in Patients With the Metabolic Syndrome. Arch. Intern. Med..

[151] Martínez-Abundis E., Drateln C.R.-V., Hernández-Salazar E., González-Ortiz M. (2007). Effect of etanercept on insulin secretion and insulin sensitivity in a randomized trial with psoriatic patients at risk for developing type 2 diabetes mellitus. Arch. Dermatol. Res..

[152] Dominguez H., Storgaard H., Rask-Madsen C., Hermann T.S., Ihlemann N., Nielsen D.B., Spohr C., Kober L., Vaag A.A., Torp-Pedersen C. (2005). Metabolic and Vascular Effects of Tumor Necrosis Factor-α Blockade with Etanercept in Obese Patients with Type 2 Diabetes. J. Vasc. Res..

[153] Dinarello C.A. (2019). The IL-1 family of cytokines and receptors in rheumatic diseases. Nat. Rev. Rheumatol..

[154] Cavalli G., Dinarello C.A. (2015). Treating rheumatological diseases and co-morbidities with interleukin-1 blocking therapies. Rheumatology.

[155] Goldbach-Mansky R. (2009). Blocking Interleukin-1 in Rheumatic Diseases. Ann. N. Y. Acad. Sci..

[156] Stassi G., De Maria R., Trucco G., Rudert W., Testi R., Galluzzo A., Giordano C., Trucco M. (1997). Nitric oxide primes pancreatic beta cells for Fas-mediated destruction in insulin-dependent diabetes mellitus. J. Exp. Med..

[157] Maedler K., Sergeev P., Ris F., Oberholzer J., Joller-Jemelka H.I., Spinas G.A., Kaiser N., Halban P.A., Donath M.Y. (2002). Glucose-induced beta cell production of IL-1beta contributes to glucotoxicity in human pancreatic islets. J. Clin. Investig..

[158] Ruscitti P., Cipriani P., Di Benedetto P., Liakouli V., Berardicurti O., Carubbi F., Ciccia F., Alvaro S., Triolo G., Giacomelli R. (2015). Monocytes from patients with rheumatoid arthritis and type 2 diabetes mellitus display an increased production of interleukin (IL)-1β via the nucleotide-binding domain and leucine-rich repeat containing family pyrin 3(NLRP3)-inflammasome activation: A Possible Implication for Therapeutic Decision in these Patients. Clin. Exp. Immunol..

[159] Marzban L., Park K., Verchere C.B. (2003). Islet amyloid polypeptide and type 2 diabetes. Exp. Gerontol..

[160] Fernández M.S. (2014). Human IAPP amyloidogenic properties and pancreatic β-cell death. Cell Calcium..

[161] Masters S.L., Dunne A., Subramanian S.L., Hull R.L., Tannahill G.M., Sharp F.A., Becker C. (2010). Activation of the NLRP3 inflammasome by islet amyloid polypeptide provides a mechanism for enhanced IL-1β in type 2 diabetes. Nat. Immunol..

[162] Park Y.J., Warnock G.L., Ao Z., Safikhan N., Meloche M., Asadi A., Kieffer T.J., Marzban L. (2017). Dual role of interleukin-1β in islet amyloid formation and its β-cell toxicity: Implications for Type 2 Diabetes and Islet Transplantation. Diabetes Metab..

[163] Böni-Schnetzler M., Boller S., Debray S., Bouzakri K., Meier D.T., Prazak R., Kerr-Conte J., Pattou F., Ehses J.A., Schuit F.C. (2009). Free fatty acids induce a proinflammatory response in islets via the abundantly expressed interleukin-1 receptor I. Endocrinology.

[164] Larsen C.M., Faulenbach M., Vaag A., Vølund A., Ehses J.A., Seifert B., Mandrup-poulsen T., Donath M.Y. (2007). Interleukin-1-receptor antagonist in type 2 diabetes mellitus. N. Engl. J. Med..

[165] Kim N.H., Kim D.L., Choi K.M., Baik S.H., Choi D.S. (2000). Serum insulin, proinsulin and proinsulin/insulin ratio in type 2 diabetic patients: As an Index of Beta-Cell Function or Insulin Resistance. Korean J. Intern. Med..

[166] Choi C.S., Kim C.H., Lee W.J., Park J.Y., Hong S.K., Lee K.U. (1999). Elevated plasma proinsulin/insulin ratio is a marker of reduced insulin secretory capacity in healthy young men. Horm. Metab. Res..

[167] van Asseldonk E.J., Stienstra R., Koenen T.B., Joosten L.A., Netea M.G., Tack C.J. (2011). Treatment with Anakinra improves disposition index but not insulin sensitivity in nondiabetic subjects with the metabolic syndrome: A Randomized, Double-Blind, Placebo-Controlled Study. J. Clin. Endocrinol. Metab..

[168] van Poppel P.C., van Asseldonk E.J., Holst J.J., Vilsbøll T., Netea M.G., Tack C.J. (2014). The interleukin-1 receptor antagonist anakinra improves first-phase insulin secretion and insulinogenic index in subjects with impaired glucose tolerance. Diabetes Obes. Metab..

[169] Hensen J., Howard C.P., Walter V., Thuren T. (2013). Impact of interleukin-1β antibody (canakinumab) on glycaemic indicators in patients with type 2 diabetes mellitus: Results of Secondary Endpoints from a Randomized, Placebo-Controlled Trial. Diabetes Metab..

[170] Cavelti-Weder C., Babians-Brunner A., Keller C., Stahel M.A., Kurz-Levin M., Zayed H., Solinger A.M., Mandrup-Poulsen T., Dinarello C.A., Donath M.Y. (2012). Effects of gevokizumab on glycemia and inflammatory markers in type 2 diabetes. Diabetes Care.

[171] Kataria Y., Ellervik C., Mandrup-Poulsen T. (2019). Treatment of type 2 diabetes by targeting interleukin-1: A Meta-Analysis of 2921 patients. Semin. Immunopathol..

[172] Giacomelli R., Ruscitti P., Alvaro S., Ciccia F., Liakouli V., Di Benedetto P., Guggino G., Berardicurti O., Carubbi F., Triolo G. (2016). IL-1β at the crossroad between rheumatoid arthritis and type 2 diabetes: May We Kill Two Birds with One Stone?. Expert Rev. Clin. Immunol..

[173] Abramson S.B., Amin A. (2002). Blocking the effects of IL-1 in rheumatoid arthritis protects bone and cartilage. Rheumatology.

[174] Strand V., Kavanaugh A.F. (2004). The role of interleukin-1 in bone resorption in rheumatoid arthritis. Rheumatology.

[175] Herder C., Dalmas E., Böni-Schnetzler M., Donath M.Y. (2015). The IL-1 Pathway in Type 2 Diabetes and Cardiovascular Complications. Trends Endocrinol. Metab..

[176] Ruscitti P., Masedu F., Alvaro S., Airò P., Battafarano N., Cantarini L., Cantatore F.P., Carlino G., D’Abrosca V., Frassi M. (2019). Anti-interleukin-1 treatment in patients with rheumatoid arthritis and type 2 diabetes (TRACK): A Multicentre, Open-Label, Randomised Controlled Trial. PLoS Med..

[177] Ruscitti P., Berardicurti O., Cipriani P., Giacomelli R. (2021). Group Ts. Benefits of anakinra versus TNF inhibitors in rheumatoid arthritis and type 2 diabetes: Long-Term Findings from Participants Furtherly Followed-Up in the Track Study, a Multicentre, Open-Label, Randomised, Controlled Trial. Clin. Exp. Rheumatol..

[178] Ruscitti P., Ursini F., Cipriani P., Greco M., Alvaro S., Vasiliki L., Di Benedetto P., Carubbi F., Berardicurti O., Gulletta E. (2019). IL-1 inhibition improves insulin resistance and adipokines in rheumatoid arthritis patients with comorbid type 2 diabetes: An Observational Study. Medicine.

[179] Unger R.H., Cherrington A.D. (2012). Glucagonocentric restructuring of diabetes: A Pathophysiologic and Therapeutic Makeover. J. Clin. Investig..

[180] Szekely Y., Arbel Y. (2018). A Review of Interleukin-1 in Heart Disease: Where Do We Stand Today?. Cardiol Ther..

[181] Ridker P.M., Everett B.M., Thuren T., MacFadyen J.G., Chang W.H., Ballantyne C., Fonseca F., Nicolau J., Koenig W., Anker S.D. (2017). Antiinflammatory Therapy with Canakinumab for Atherosclerotic Disease. N. Engl. J. Med..

[182] Everett B.M., Cornel J.H., Lainscak M., Anker S.D., Abbate A., Thuren T., Libby P., Glynn R.J., Ridker P.M. (2019). Anti-Inflammatory Therapy With Canakinumab for the Prevention of Hospitalization for Heart Failure. Circulation.

[183] Kenny H.C., Abel E.D. (2019). Heart Failure in Type 2 Diabetes Mellitus. Circ. Res..

[184] Everett B.M., Donath M.Y., Pradhan A.D., Thuren T., Pais P., Nicolau J., Glynn R.J., Libby P., Ridker P.M. (2018). Anti-Inflammatory Therapy With Canakinumab for the Prevention and Management of Diabetes. J. Am. Coll. Cardiol..

[185] Rehman K., Akash M.S.H., Liaqat A., Kamal S., Qadir M.I., Rasul A. (2017). Role of Interleukin-6 in Development of Insulin Resistance and Type 2 Diabetes Mellitus. Crit. Rev. Eukaryot. Gene Expr..

[186] Emanuelli B., Peraldi P., Filloux C., Chavey C., Freidinger K., Hilton D.J., Hotamisligil G.S., Van Obberghen E. (2001). SOCS-3 Inhibits Insulin Signaling and Is Up-regulated in Response to Tumor Necrosis Factor-α in the Adipose Tissue of Obese Mice. J. Biol. Chem..

[187] Ogata A., Morishima A., Hirano T., Hishitani Y., Hagihara K., Shima Y., Narazaki M., Tanaka T. (2011). Improvement of HbA1c during treatment with humanised anti-interleukin 6 receptor antibody, tocilizumab: Figure. Ann. Rheum. Dis..

[188] Otsuka Y., Kiyohara C., Kashiwado Y., Sawabe T., Nagano S., Kimoto Y., Ayano M., Mitoma H., Akahoshi M., Arinobu Y. (2018). Effects of tumor necrosis factor inhibitors and tocilizumab on the glycosylated hemoglobin levels in patients with rheumatoid arthritis; an observational study. PLoS ONE.

[189] Kohase M., May L.T., Tamm I., Vilcek J., Sehgal P.B. (1987). A cytokine network in human diploid fibroblasts: Interactions of Beta-Interferons, Tumor Necrosis Factor, Platelet-Derived Growth Factor, and Interleukin-1. Mol. Cell. Biol..

[190] Loppnow H., Libby P. (1990). Proliferating or interleukin 1-activated human vascular smooth muscle cells secrete copious interleukin. J. Clin. Investig..

[191] Sironi M., Breviario F., Proserpio P., Biondi A., Vecchi A., Van Damme J., Dejana E., Mantovani A. (1989). IL-1 stimulates IL-6 production in endothelial cells. J. Immunol..

[192] Tosato G., Jones K.D. (1990). Interleukin-1 induces interleukin-6 production in peripheral blood monocytes. Blood.

[193] Le J.M., Vilcek J. (1989). Interleukin 6: A Multifunctional Cytokine Regulating Immune Reactions and the Acute Phase Protein Response. Lab. Investig..

[194] Castell J.V., Gómez-Lechón M.J., David M., Fabra R., Trullenque R., Heinrich P.C. (1990). Acute-phase response of human hepatocytes: Regulation of Acute-Phase Protein Synthesis by Interleukin-6. Hepatology..

[195] Blair H.A., Deeks E.D. (2017). Abatacept: A Review in Rheumatoid Arthritis. Drugs.

[196] Herrero-Beaumont G., Martínez Calatrava M.J., Castañeda S. (2012). Abatacept mechanism of action: Concordance with its Clinical Profile. Reumatol. Clin..

[197] Ursini F., Mauro D., Naty S., Gagliardi D., Grembiale R.D. (2012). Improvement in insulin resistance after short-term treatment with abatacept: Case Report and Short Review. Clin. Rheumatol..

[198] Ursini F., Russo E., Letizia Hribal M., Mauro D., Savarino F., Bruno C., Tripolino C., Rubino M., Naty S., Grembiale R.D. (2015). Abatacept improves whole-body insulin sensitivity in rheumatoid arthritis: An Observational Study. Medicine.

[199] Lee B.C., Lee J. (2014). Cellular and molecular players in adipose tissue inflammation in the development of obesity-induced insulin resistance. Biochim. Biophys. Acta.

[200] Kintscher U., Hartge M., Hess K., Foryst-Ludwig A., Clemenz M., Wabitsch M., Fischer-Posovszky P., Barth T.F.E., Dragun D., Skurk T. (2008). T-lymphocyte infiltration in visceral adipose tissue: A Primary Event in Adipose Tissue Inflammation and the Development of Obesity-Mediated Insulin Resistance. Arterioscler Thromb. Vasc. Biol..

[201] Nishimura S., Manabe I., Nagasaki M., Eto K., Yamashita H., Ohsugi M., Otsu M., Hara K., Ueki K., Sugiura S. (2009). CD8+ effector T cells contribute to macrophage recruitment and adipose tissue inflammation in obesity. Nat. Med..

[202] Pollack R.M., Donath M.Y., LeRoith D., Leibowitz G. (2016). Anti-inflammatory Agents in the Treatment of Diabetes and Its Vascular Complications. Diabetes Care.

[203] Izumi K., Murata O., Higashida-Konishi M., Kaneko Y., Oshima H., Takeuchi T. (2021). Steroid-Sparing Effect of Tocilizumab and Methotrexate in Patients with Polymyalgia Rheumatica: A Retrospective Cohort Study. J. Clin. Med..

[204] Hwang J.L., Weiss R.E. (2014). Steroid-induced diabetes: A clinical and molecular approach to understanding and treatment. Diabetes/Metab. Res. Rev..

[205] Ito S., Ogishima H., Kondo Y., Sugihara M., Hayashi T., Chino Y., Goto D., Matsumoto I., Sumida T. (2014). Early diagnosis and treatment of steroid-induced diabetes mellitus in patients with rheumatoid arthritis and other connective tissue diseases. Mod. Rheumatol..

[206] Beaupere C., Liboz A., Fève B., Blondeau B., Guillemain G. (2021). Molecular Mechanisms of Glucocorticoid-Induced Insulin Resistance. Int. J. Mol. Sci..

[207] Apostolopoulos D., Morand E.F. (2017). It hasn’t gone away: The Problem of Glucocorticoid Use in Lupus Remains. Rheumatology.

[208] Smolen J.S., Landewé R.B.M., Bijlsma J.W.J., Burmester G.R., Dougados M., Kerschbaumer A., McInnes I.B. (2020). Eular recommendations for the management of rheumatoid arthritis with synthetic and biological disease-modifying antirheumatic drugs: 2019 Update. Ann. Rheum. Dis..

[209] Ahlqvist E., Storm P., Käräjämäki A., Martinell M., Dorkhan M., Carlsson A., Vikman P., Prasad R.B., Aly D.M., Almgren P. (2018). Novel subgroups of adult-onset diabetes and their association with outcomes: A Data-Driven Cluster Analysis of Six Variables. Lancet Diabetes Endocrinol..

[210] Anjana R.M., Baskar V., Nair A.T.N., Jebarani S., Siddiqui M.K., Pradeepa R., Unnikrishnan R., Palmer C., Pearson E., Mohan V. (2020). Novel subgroups of type 2 diabetes and their association with microvascular outcomes in an Asian Indian population: A Data-Driven Cluster Analysis: The INSPIRED Study. BMJ Open Diabetes Res. Care.

[211] Wright L.A., Hirsch I.B. (2017). Metrics Beyond Hemoglobin A1C in Diabetes Management: Time in Range, Hypoglycemia, and Other Parameters. Diabetes Technol. Ther..

[212] Ajjan R.A. (2017). How Can We Realize the Clinical Benefits of Continuous Glucose Monitoring?. Diabetes Technol. Ther..

[213] Amigues I., Pearlman A.H., Patel A., Reid P., Robinson P.C., Sinha R., Kim A.H., Youngstein T., Jayatilleke A., Konig M. (2020). Coronavirus disease 2019: Investigational Therapies in the Prevention and Treatment of Hyperinflammation. Expert Rev. Clin. Immunol..

[214] Goodarzi P., Mahdavi F., Mirzaei R., Hasanvand H., Sholeh M., Zamani F., Sohrabi M., Tabibzadeh A., Jeda A.S., Niya M.H.K. (2020). Coronavirus disease 2019 (COVID-19): Immunological Approaches and Emerging Pharmacologic Treatments. Int. Immunopharmacol..

[215] Mangalmurti N., Hunter C.A. (2020). Cytokine Storms: Understanding COVID-19. Immunity.

[216] Pinheiro M.M., Fabbri A., Infante M. (2021). Cytokine storm modulation in COVID-19: A Proposed Role for Vitamin D and DPP-4 Inhibitor Combination Therapy (VIDPP-4i). Immunotherapy.

[217] Cauchois R., Koubi M., Delarbre D., Manet C., Carvelli J., Blasco V.B., Jean R., Fouche L., Bornet C., Pauly V. (2020). Early IL-1 receptor blockade in severe inflammatory respiratory failure complicating COVID-19. Proc. Natl. Acad. Sci. USA.

[218] Pasin L., Cavalli G., Navalesi P., Sella N., Landoni G., Yavorovskiy A.G., Likhvantsev V.V., Zangrillo A., Dagna L., Monti G. (2021). Anakinra for patients with COVID-19: A Meta-Analysis of Non-Randomized Cohort Studies. Eur. J. Intern. Med..

[219] Group R.C. (2021). Tocilizumab in patients admitted to hospital with COVID-19 (Recovery): A Randomised, Controlled, Open-Label, Platform Trial. Lancet.

[220] Hasan M.J., Rabbani R., Anam A.M., Huq S.M.R., Polash M.M.I., Nessa S.S.T., Bachar S.C. (2021). Impact of high dose of baricitinib in severe COVID-19 pneumonia: A Prospective Cohort Study in Bangladesh. BMC Infect. Dis..

[221] Saghir S.A.M., AlGabri N.A., Alagawany M.M., Attia Y.A., Alyileili S.R., Elnesr S.S., Shafi M.E., Al-Shargi O.Y., Al-Balagi N., Alwajeeh A.S. (2021). Chloroquine and Hydroxychloroquine for the Prevention and Treatment of COVID-19: A Fiction, Hope or Hype? An Updated Review. Ther. Clin. Risk Manag..

[222] Infante M., Ricordi C., Alejandro R., Caprio M., Fabbri A. (2020). Hydroxychloroquine in the COVID-19 pandemic era: In Pursuit of a Rational Use for Prophylaxis of SARS-CoV-2 Infection. Expert Rev. Anti Infect. Ther..

[223] Drucker D.J. (2020). Coronavirus Infections and Type 2 Diabetes-Shared Pathways with Therapeutic Implications. Endocr. Rev..

[224] Noor F.M., Islam M.M. (2020). Prevalence and Associated Risk Factors of Mortality Among COVID-19 Patients: A Meta-Analysis. J. Community Health.

[225] Paoli A., Gorini S., Caprio M. (2020). The dark side of the spoon—Glucose, ketones and COVID-19: A Possible Role for Ketogenic Diet?. J. Transl. Med..

[226] Drucker D.J. (2021). Diabetes, obesity, metabolism, and SARS-CoV-2 infection: The End of the Beginning. Cell Metab..

[227] Schreiber K., Hendricks O. (2021). First data on COVID-19 morbidity and mortality in patients with rheumatic disease from South Korea. Lancet Rheumatol..

[228] Ahmed S., Gasparyan A.Y., Zimba O. (2021). Comorbidities in rheumatic diseases need special consideration during the COVID-19 pandemic. Rheumatol. Int..

